# Prediction of clusters of miRNA binding sites in mRNA candidate genes of breast cancer subtypes

**DOI:** 10.7717/peerj.8049

**Published:** 2019-11-13

**Authors:** Dana Aisina, Raigul Niyazova, Shara Atambayeva, Anatoliy Ivashchenko

**Affiliations:** Department of Biotechnology, SRI of Biology and Biotechnology Problems, Al-Farabi Kazakh National University, Almaty, Kazakhstan

**Keywords:** Binding site, Cluster, Gene, Breast cancer, miRNA

## Abstract

The development of breast cancer (BC) subtypes is controlled by distinct sets of candidate genes, and the expression of these genes is regulated by the binding of their mRNAs with miRNAs. Predicting miRNA associations and target genes is thus essential when studying breast cancer. The MirTarget program identifies the initiation of miRNA binding to mRNA, the localization of miRNA binding sites in mRNA regions, and the free energy from the binding of all miRNA nucleotides with mRNA. Candidate gene mRNAs have clusters (miRNA binding sites with overlapping nucleotide sequences). mRNAs of *EPOR, MAZ* and *NISCH* candidate genes of the HER2 subtype have clusters, and there are four clusters in mRNAs of *MAZ, BRCA2* and *CDK6* genes. Candidate genes of the triple-negative subtype are targets for multiple miRNAs. There are 11 sites in *CBL* mRNA, five sites in *MMP2* mRNA, and *RAB5A* mRNA contains two clusters in each of the three sites. In *SFN* mRNA, there are two clusters in three sites, and one cluster in 21 sites. Candidate genes of luminal A and B subtypes are targets for miRNAs: there are 21 sites in *FOXA1* mRNA and 15 sites in* HMGA2* mRNA. There are clusters of five sites in mRNAs of *ITGB1* and *SOX4* genes. Clusters of eight sites and 10 sites are identified in mRNAs of *SMAD3* and *TGFB1* genes, respectively. Organizing miRNA binding sites into clusters reduces the proportion of nucleotide binding sites in mRNAs. This overlapping of miRNA binding sites creates a competition among miRNAs for a binding site. From 6,272 miRNAs studied, only 29 miRNAs from miRBase and 88 novel miRNAs had binding sites in clusters of target gene mRNA in breast cancer. We propose using associations of miRNAs and their target genes as markers in breast cancer subtype diagnosis.

## Introduction

Breast cancer (BC) is the most common cancer in women worldwide, and the second most common cancer overall. Statistics indicate an intense, steady increase in the incidence and mortality of BC among women (Benson & Jatoi, 2012). More than 50% of BC cases are first detected in the late stages of the disease. Every year, 1,400,000 new cases of the disease are diagnosed globally (Jemal et al., 2010). Establishing the relationship between the miRNAs and mRNA genes involved in the development of BC (candidate genes) is a promising area of research. miRNAs are found in tumors and blood, and may be potential biomarkers of BC (Adhami et al., 2018; Hannafon et al., 2016; Kurozumi et al., 2017; Lagendijk et al., 2018; McDermott et al., 2014; Piasecka et al., 2018; Zhang et al., 2017). Several studies have been devoted to establishing a correlation between the expression of miRNA and various BC subtypes (Biagioni et al., 2012; Blenkiron et al., 2007; Enerly et al., 2011; Lee et al., 2013; Lowery et al., 2009; Mattie et al., 2006; Telonis et al., 2015; Yang et al., 2017). Disruptions in the regulation of miRNA expression can regulate the expression of oncogenes and oncosuppressors, thus affecting the development of a tumor. An increase or decrease in the expression of certain miRNAs influence the onset and progression of a tumor (Wang et al., 2013). Some miRNAs are overexpressed if the expression of candidate genes in both benign and malignant tumors is significantly lowered (Tahiri et al., 2014). It has been shown that many intron miRNAs are expressed together with host genes. Changes in miRNA expression may be associated with chromosomal mutations (Qian et al., 2012), epigenetic modifications (Yu et al., 2013) or biogenesis defects (Sung et al., 2012). miRNAs that inhibit the translation of tumor suppressor and apoptosis gene mRNA function as oncogenes, which contributes to oncogenesis (Wu et al., 2013). Other miRNAs may be tumor suppressors if their target genes are oncogenes and cell cycle genes (Nian et al., 2013). Presently, there is little information about miRNA and genes involved in BC subtypes. Therefore, in the present work, the association between the miRNAs and mRNAs of candidate genes of BC subtypes are revealed. According to miRBase, more than 90% of miRNA have a length in the range of 20–25 nucleotides (http://mirbase.org). Similar to the length of primers in the polymerase chain reaction, miRNA nucleotide sequence length is necessary for the selective interaction with mRNA (Huggett & O’Grady, 2014). One miRNA can have binding sites (BSs) in the mRNA of many genes (Atambayeva et al., 2017; Ivashchenko et al., 2016; Niyazova et al., 2015), and the mRNA of one gene can have BSs for many miRNAs (Kondybayeva et al., 2018).

In this article, we show the advantage of utilizing the interaction between miRNA and mRNA when studying the binding of miRNA with the mRNA of BC subtype candidate genes, which contain clusters of miRNA BSs.

## Materials & Methods

The nucleotide (nt) sequences of candidate genes of BC subtypes were downloaded from GenBank (http://www.ncbi.nlm.nih.gov). These specific candidate genes are responsible for the development of triple-negative subtype, luminal A and B subtypes, and HER2 subtype of BC (Table S1). Information about miRNAs that presumably bind to candidate genes of BC is provided in Table S2. The table indicates that the miRNAs studied are present in blood, serum, plasma, and cells in BC or other types of cancer. The nucleotide sequences of mRNA genes of *Chlorocebus sabaeus*—Csa, *Gorilla gorilla*—Ggo, *Homo sapience*—Hsa, *Macaca mulatta*—Mml, *Mus musculus*—Mmu, *Pan paniscus*—Ppa, *Pan troglodytes*—Ptr, *Papio Anubis*—Pan, *Pongo abelii*—Pab, and *Rattus norvegicus—Rno* were downloaded from NCBI (http://www.ncbi.nlm.nih.gov). The nucleotide sequences of 2,565 miRNAs were taken from miRBase, and 3,707 miRNAs from a previous study (Londin et al., 2015). The Reads Per Kilobase Million (RPKM) value (Mortazavi et al., 2008) was given in the Human Protein Atlas data (https://www.proteinatlas.org/ENSG00000150093-ITGB1/tissue/breast). Human Protein Atlas data were used as a quantitative measure of breast transcript expression.

The miRNA BSs in 5′UTRs, CDSs and 3′UTRs of several genes were predicted using the MirTarget program (Ivashchenko et al., 2016; Ivashchenko, Issabekova & Berillo, 2013). This program defines the following features of miRNA binding to mRNAs: (a) the start of the initiation of miRNA binding to mRNAs; (b) the localization of miRNA BSs in 5′UTRs, CDSs and 3′UTRs of the mRNAs; (c) the free energy of interaction between miRNA and the mRNA (ΔG, kJ/mole); and (d) the schemes of nucleotide interactions between miRNAs and mRNAs. The ratio ΔG/ΔGm (%) was determined for each site (ΔGm equals the free energy of miRNA binding with its fully complementary nucleotide sequence). The miRNA BSs located in mRNAs had ΔG/ΔGm ratios of 87% or more. ΔG/ΔGm ratios were taken on the assumption that the members of the miRNA of one family differ by generally no more than one to three nucleotides and, along with a miRNA length of 22 nt, the ΔG/ΔGm value was 96% (21 nt/22 nt = 96%) − 87% (19 nt/22 nt = 87%). With a larger difference in the number of mismatched nucleotides, the probability of two or more miRNAs binding in one site increases, despite the miRNA’s natural ability to interact selectively with the mRNA of the target gene. The MirTarget program identifies the positions of the BSs on the mRNA, beginning with the first nucleotide of the mRNA’s 5′UTR. The MirTarget program found hydrogen bonds between adenine (A) and uracil (U), guanine (G) and cytosine (C), G and U, and A and C. The distance between A and C was 1.04 nanometers; 1.03 nanometers between G and C, and A and U; and 1.02 nanometers between G and U (Leontis, Stombaugh & Westhof, 2002). The numbers of hydrogen bonds in the G-C, A-U, G-U and A-C interactions were found to be 3, 2, 1 and 1, respectively (Kool, 2001; Lemieux & Major, 2002; Leontis, Stombaugh & Westhof, 2002). The characteristics of the interaction between miRNA and mRNA reflect the intermolecular interactions of their molecules and were calculated for given parameters without their variation. Consequently, the results have no statistical scatter. Other factors that may have influenced these interactions have not been studied. Our article does not address the subject of changing the concentration ratio of miRNA and mRNA, because this aspect is of independent interest and is not part of the objectives of this work. For any other pathology, other candidate genes should be used, and other miRNA BSs should be determined.

The MirTarget program determines single miRNA BSs in mRNA, and miRNA BSs in clusters (arranged in series with overlapping of nucleotide sequences of the same or several miRNAs). In this study we hypothesized that miRNA BSs in mRNA are organized into clusters. The MirTarget program does not work directly with miRBase and NCBI databases. The searches for target genes from 17,508 human genes in a special format from NCBI, for the known miRNAs from miRBase, and for novel miRNAs from other sources will be available on request at mirtarget8@gmail.com.

## Results

The adequate prediction of miRNA BSs in mRNA target genes is a key problem when studying the biological role of miRNA in the regulation of gene expression. We have developed a MirTarget program that predicts the BSs of miRNA using mRNA, thereby revealing new properties of miRNA. Before presenting the results, we will provide a few specific examples that demonstrate the features of the MirTarget program.

The schemes of miRNA nucleotide interaction with mRNA BSs are shown in Figs. S1–S3. The following advantages of the MirTarget program are shown: (a) almost all miRNA nucleotides interact with mRNA; (b) the formation of non-canonical pairs G-U and A-C do not change the double-stranded arrangement of the miRNA complex with mRNA since the distances between G-U and A-C are equal to the distances between G-C and A-U; (c) the free energy of interaction is an important criterion for binding miRNA to mRNA; and (d) the localization of the miRNA BSs in 5′UTR, CDS and 3′UTR.

Several miRNAs were bound to the entire nucleotide sequence of mRNA of triple-negative BC subtype candidate genes (Fig. S1). For example, miR-5095, miR-5096, miR-619-5p, miR-1273g-3p, and miR-1273f with entire nucleotide sequences were bound to the mRNA of the *ATM* gene. MiR-5095, miR-1273e, miR-1273f were bound to the mRNA *IL11* gene by all nucleotides. Similarly, miR-1273c and miR-1285-3p were bound to the mRNA of the *STMN1* gene, and ID00436.3p-miR was bound with the mRNA of the *SFN* gene.

ID01810.3p-miR had BSs in 5′UTR of the mRNA *CBL* gene (Fig. S1). Of the 23 nucleotides in ID01810.3p-miR, only one nucleotide could not form hydrogen bonds with mRNAs, and the other nucleotides formed a double-stranded helical structure with mRNA. Three pairs of G-U and three pairs of A-C, each with one hydrogen bond, contributed to the preservation of this structure due to the stacking interactions between adjacent bases (Yakovchuk, Protozanova & Frank-Kamenetskii, 2006). The binding of ID01810.3p-miR to the mRNA of the *CBL* gene caused non-canonical pairs G-U and A-C to form, and there was no interaction between A and G in the second position. Despite this, the free energy of the interaction of ID01810.3p-miR with the mRNA of the *CBL* gene was 87% of the maximum. The interaction schemes of ID01321.5p-miR with mRNA *RUNX1* gene, miR-3198 with mRNA *CBL* gene, miR-1273d with mRNA *IL11* gene, and miR-5585-3p with mRNA *STMN1* gene are shown in Fig. S1. The free energy of interaction of these pairs of miRNAs and mRNAs was 87–98% of the maximum value of ΔGm (Table 1).

**Table 1:** Characteristics of miRNA interaction in the mRNA of BC subtype triple-negative.

Gene, RPKM	miRNA	Start of binding site, nt	ΔG, kJ/mole	ΔG/ΔGm, %	Length, nt
*CBL*	ID03332.3p-miR (4)	16 ÷ 25	−134 ÷−140	90 ÷ 94	24
3.9	ID01310.3p-miR (4)	17 ÷ 26	−121	92	22
	ID02761.3p-miR	28	−138	93	24
	miR-1908-3p	30	−121	92	21
	ID00278.3p-miR	32	−125	91	23
	ID02430.3p-miR	34	−110	98	18
*MMP2*	ID00278.3p-miR	110	−123	89	23
192.4	ID01310.3p-miR	113	−121	92	22
	ID03037.3p-miR	115	−121	90	22
	ID03345.5p-miR	124	−127	90	24
	ID03368.3p-miR	125	−117	89	23
*RAB5A*	ID02930.3p-miR	184	−132	89	24
16.1	ID03445.3p-miR	189	−127	90	24
	ID01859.5p-miR	191	−121	89	23
	ID01804.3p-miR	325	−140	88	25
	ID03367.5p-miR	328	−121	97	20
	ID00061.3p-miR	334	−127	92	22
ATM**	ID03006.5p-miR	9,778	−121	89	24
3.9	miR-5095	9,787	−108	93	21
	miR-619-5p	9,793	−119	98	22
	miR-1273a	11,054	−119	90	25
	miR-1273g-3p	11,076	−113	96	21
	ID00367.5p-miR	11,069	−110	90	22
CBL**	miR-1273a	7,727	−117	89	25
	miR-1273g-3p	7,749	−115	98	21
3.9	ID01838.5p-miR	7,728	−117	93	24
IL11**	miR-1273f	1,466	−102	98	19
0.1	miR-1273d	1,467	−121	89	25
	miR-1273e	1,476	−113	96	22
	ID01404.5p-miR	1,470	−113	91	23
RUNX1**	miR-466 (2)	5,456 ÷ 5,460	−106 ÷ -110	91 ÷ 95	23
9.0	ID01030.3p-miR (2)	5,454 ÷ 5,464	−108 ÷−113	89 ÷ 93	23
	D00436.3p-miR	5,464	−108	93	23
SFN**	miR-6089	826	−129	87	24
9.4	ID01774.5p-miR	835	−129	90	23
	miR-6846-5p	839	−113	91	22
	ID00790.3p-miR	1,179	−104	89	23
	ID02868.3p-miR	1,188	−113	90	23
	miR-466 (6)	1,190 ÷ 1,200	−106	91	23
	ID01030.3p-miR (6)	1,190 ÷ 1,200	−108	89	23
	ID00436.3p-miR (7)	1,190 ÷ 1,202	−104	89	23
	ID01727.5p-miR (2)	1,203 ÷ 1,205	−104 ÷−106	89 ÷ 91	23
	ID02882.3p-miR	1,210	−108	91	21
STMN1**	miR-1273a	1,729	−115	87	25
	miR-1273g-3p	1,751	−108	93	21
	ID00367.5p-miR	1,744	−113	91	22
6.6	ID03011.5p-miR	1,730	−106	91	22

**Notes.**

Genes without *—miRNA binding sites are in the 5′UTR, genes with *—miRNA binding sites are in the CDS, **—miRNA binding sites are in the 3′UTR; values in parentheses indicate the number of binding sites; ÷—the change of the parameter in the interval. Different repeating clusters of miRNA binding sites in different genes are coloured.

With the mRNA of luminal A and B BC subtype candidate genes, several miRNAs of the entire nucleotide sequences have been associated (Fig. S2). For example, ID01403.5p-miR and ID02428.3p-miR were bound to the mRNA *HMGA2* gene. ID03332.3p-miR bound to the mRNA *FOXA1* gene, and ID01593.5p-miR was fully complementarily bound to the mRNA *ANGPTL4* gene.

MiR-3960, miR-7111-3p and ID01352.3p-miR were bound to the mRNA *MAZ* gene by all nucleotides (Fig. S3). MiR-877-3p was bound by all nucleotides to the mRNA of *NISCH* and *MAZ* genes, and ID00436.3p-miRwas bound to the mRNA of the *CDK6* gene.

## Subtype triple-negative breast cancer

The *CBL* gene is a target for six miRNAs, two of which have four BSs (Table 1). A cluster of 12 BSs for six miRNAs was located from 16 nt to 55 nt. All BSs for miRNAs had a total length of 270 nt. The cluster size was 40 nt, with a length of 142 nt for 5′UTR of the mRNA *CBL* gene, so there was a clear need for cluster organization of miRNA BSs. The BSs were compacted 6.8 times. The average free energy of interaction between the six miRNAs with the mRNA of the *CBL* gene was −127 kJ/mole.

**Figure 1:**
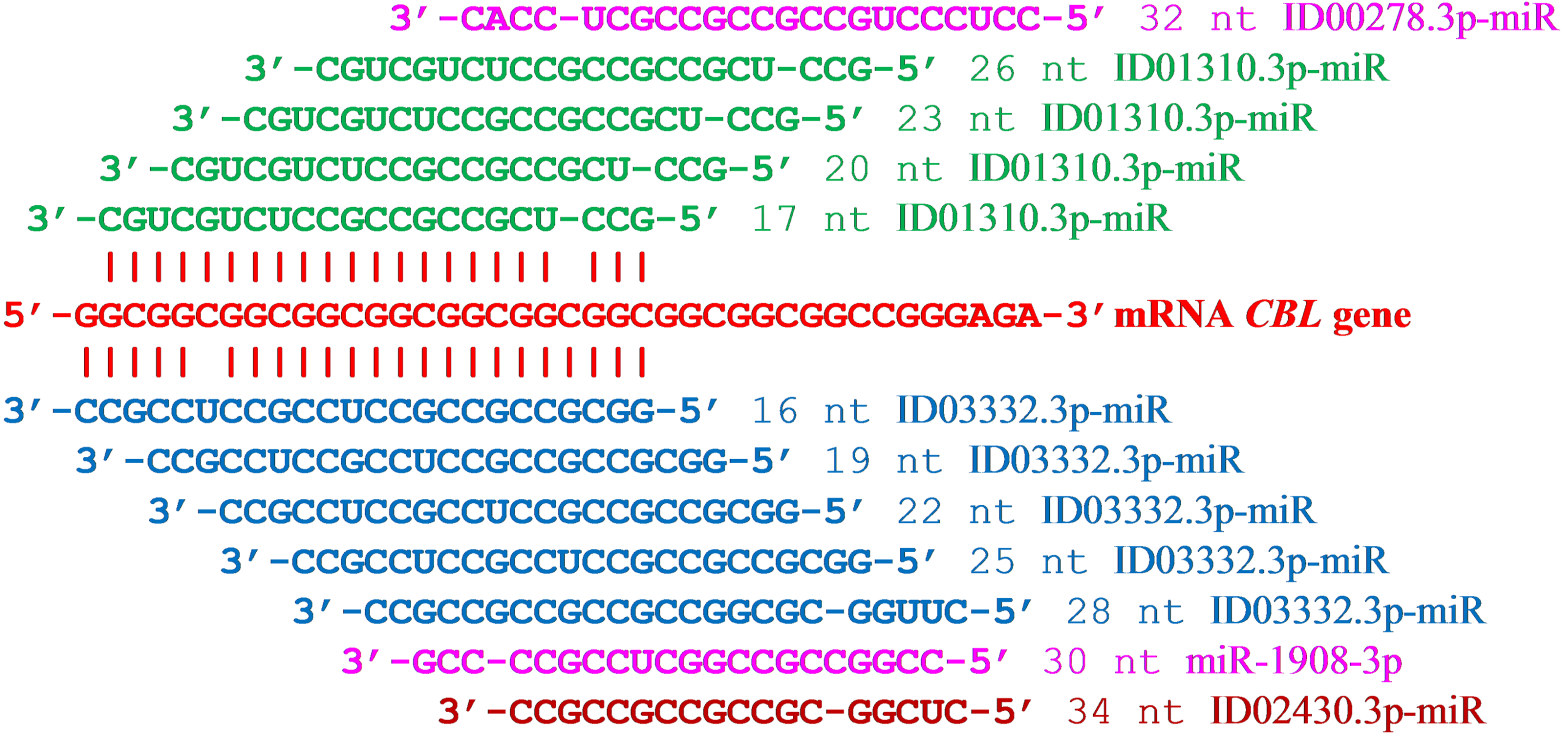
Location of nucleotide sequences of miRNA binding sites cluster in mRNA *CBL* gene.

**Figure 2:**
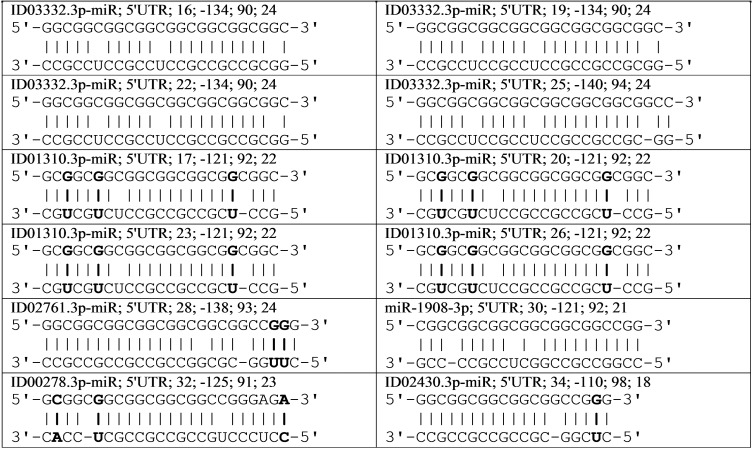
Schemes of interactions of miRNAs with mRNA of *CBL* gene in cluster of binding sites. miRNA; the miRNA region; start of binding site (nt); the free energy, ΔG (kJ/mole); the ΔG/ΔGm (%); length of miRNA (nt). The upper and lower nucleotide sequences of mRNA and miRNA, respectively. The nucleotides of non-canonical pairs G–U and A–C highlighted in bold type.

The results of the supposed interactions of six miRNAs with the mRNA *CBL* gene can be represented as a diagram showing the location of the miRNA BSs relative to the mRNA cluster (Fig. 1). A feature of ID03332.3p-miR is the starting location of their repeating BSs across three nucleotides. This miRNA interacts with mRNA by displacing its BSs, coinciding with the open reading frame of the mRNA *CBL* gene. The schemes of interaction among these 12 miRNAs with the mRNA of the *CBL* gene are shown in Fig. 2. It can be seen from these schemes that the interaction between non-canonical pairs A-C and G-U increases the stability of the binding of miRNA to mRNA. From the data presented (Fig. 1) it can be seen that no more than one miRNA can bind with a cluster, which causes competition between the miRNAs to bind to the mRNA of a target gene. Some genes expressed in the mammary gland with an RPKM value of less than 10 contain repeats of nucleotides that are targeted by several miRNAs. In the mRNA *CBL* gene, which has a RPKM value of 3.9, four BSs were identified for ID03332.3p-miR and ID01310.3p-miR in a cluster located at 5′UTR from 16 nt to 54 nt (Table 1). Another example of a target gene for miRNA with nucleotide repeats in 3′UTR is the *SFN* gene with an RPKM value of 9.4 (Table 1). miR-466, ID01030.3.3p-miR and ID00436.3p-miR each had six BSs in the cluster from 1,190 nt to 1,214 nt.

Five miRNAs with overlapping BSs were found in the 5′UTR of the mRNA *MMP2* gene with a cluster length of 39 nt. The total length of miRNA was 114 nt, which is 2.9 times greater than the total length of a cluster. The average free energy of interaction among the five miRNAs with the mRNA of *MMP2* gene was −122 kJ/mole.

The *RAB5A* gene was a target for six miRNAs, and their BSs were formed into two clusters (Table 1). The cluster length of BSs for ID02930.3p-miR, ID03445.3p-miR, and ID01859.5p-miR located from 184 nt to 214 nt was 31 nt. The total length of the BSs of these miRNAs, arranged in succession, was 71 nt. Due to the overlapping of nucleotide sequences of the BSs of these miRNAs, the total length of the BSs was 2.3 times lower. However, at the same time, only one miRNA can interact with mRNA in the 31 nt segment. Thus, there is a competition between the three miRNAs to bind to the mRNA of the target gene. It is more likely that miRNA will bind with a greater free energy of interaction with mRNA at equal concentrations, or miRNA will be present in greater concentrations at an equal free energy of interaction with mRNA. The second cluster of miRNA BSs was located from 325 nt to 356 nt. The total length of miRNA BSs was 2.1 times the length of the cluster. The average free energy of interaction of the six miRNAs with the mRNA of *RAB5A* was −128 kJ/mole.

There were six miRNA BSs that formed two clusters of BSs in the mRNA of the *ATM* gene (Table 1). The total miRNA lengths for the first and second cluster were 67 and 68 nt, respectively. The decrease in the total length of the miRNA BSs at the overlap of their nucleotide sequence clusters was 1.6–1.8 times. The average free energy of interaction among the six miRNAs with the mRNA of *ATM* gene was −115 kJ/mole.

The cluster of BSs in the mRNA of the *IL11* gene was located from 1466 nt to 1498 nt with a length of 31 nt. The sum of the lengths of BSs was 2.9 times the length of the cluster.

The BS clusters for three miRNAs were identified in 3′UTR of mRNA *RUNX1* and *CBL* genes. Compacting the length of the BSs of miRNAs created competition between them for the BS in mRNA. The average free energy of the interactions between miRNA and mRNA in *CBL* and *RUNX1* gene clusters were −116 kJ/mole and −109 kJ/mole, respectively.

There were two clusters of BSs for three miRNAs from 826 nt to 861 nt, and another cluster from 1,179 nt to 1,231 nt in 3′UTR of the mRNA *SFN* gene. The sum of the lengths of all miRNA BSs of the two clusters was 619 nt. Due to the clustering of BSs of these miRNAs, the actual BS was only 89 nt long, which is seven times less than the length of 3′UTR of the mRNA *SFN* gene, which is 498 nt. The average free energy of miRNA binding at 27 sites was −108 kJ/mole.

The *STMN1* gene was the target of four miRNAs, its BSs in 3′UTR were 43 nt, while the total miRNA length was 90 nt. The average free energy of miRNA binding at four sites was −110 kJ/mole.

The free energy value was higher than −125 kJ/mole for the interactions among ID03332.3p-miR, ID02430.3p-miR, ID02761.3p-miR, ID00278.3p-miR, ID03345.5p-miR, ID02930.3p-miR, ID03445.3p-miR, ID01804.3p-miR, ID00061.3p-miR, ID03006.5p-miR, miR-1273d, miR-6089 and ID01774.5p-miR with the mRNAs of *CBL, MMP2, RAB5A, ATM, IL11* and *SFN* genes.

### Subtype luminal A and B breast cancer

Eighteen miRNA BSs with overlapping nucleotide sequences were identified in 5′UTR of the mRNA of the *FOXA1* gene (Table 2). Twenty BSs formed a cluster with a length of 52 nt, spanning from 99 nt to 151 nt. The total length of all 20 BSs was 447 nt, which was longer than 5′UTR with a length of 312 nt. Since the cluster length was 52 nt, only two miRNAs could be bound simultaneously, and other miRNAs did not affect the expression of the *FOXA1* gene.

**Table 2:** Characteristics of miRNAs interaction in mRNA of BC subtype luminal A and B.

Gene, RPKM	miRNA	Start of binding site, nt	ΔG, kJ/mole	ΔG/ΔGm, %	Length, nt
*FOXA1*	ID00297.5p-miR	99	−123	89	24
10.2	ID02106.3p-miR	110	−123	89	23
	ID00252.5p-miR	111	−140	94	24
	ID02769.5p-miR	112	−127	92	22
	ID01099.5p-miR	116	−108	100	17
	ID01190.5p-miR	118	−108	100	17
	ID00296.3p-miR	115	−140	89	25
	ID01641.3p-miR	122	−134	90	24
	ID01403.5p-miR	120	−123	91	23
	ID00061.3p-miR	127	−129	94	22
	ID03367.5p-miR (2)	121 ÷ 122	−117	93	20
	miR-3960	120	−115	92	20
	ID00071.3p-miR (2)	118 ÷ 121	−117 ÷−121	93 ÷ 97	20
	ID02457.3p-miR	118	−108	100	17
	ID02595.5p-miR	118	−115	92	20
	ID01702.3p-miR	120	−140	89	25
	ID00457.3p-miR	124	−123	91	22
	ID02499.3p-miR (2)	127 ÷ 130	−119 ÷−121	92 ÷ 93	21
*HMGA2*	miR-6756-5p	529	−117	87	23
0.0	ID01737.3p-miR	539	−119	93	21
	ID01041.5p-miR (2)	541 ÷ 544	−129 ÷−134	88 ÷ 91	24
	ID00089.3p-miR	542	−125	91	22
	ID01323.3p-miR	542	−117	95	20
	ID02296.5p-miR	542	−115	93	20
	ID0296.3p-miR	544	−146	93	25
	ID01641.3p-miR	544	−142	96	24
	ID01403.5p-miR	547	−119	88	23
	ID00061.3p-miR	550	−132	95	22
	ID03367.5p-miR	550	−115	92	20
	miR-3960	549	−117	93	20
	miR-4739	573	−123	87	25
	ID00425.5p-miR	575	−121	88	24
	ID00564.5p-miR	585	−110	90	22
ITGB1*	ID02187.5p-miR	91	−127	92	23
63.6	miR-4787-5p	92	−123	92	22
	ID00457.3p-miR	95	−123	91	22
	ID02770.5p-miR	98	−117	93	20
	ID01184.3p-miR	101	−117	93	20
HMGA2**	ID01970.3p-miR	1,255	−113	90	23
0.0	ID00849.3p-miR (2)	1,261 ÷ 1,268	−117	90	22
	ID01545.3p-miR	1,275	−115	95	21
SMAD3**	miR-4690-5p	2,066	−115	92	22
	ID02822.5p-miR	2,070	−127	91	23
	ID00978.5p-miR	2,072	−119	90	22
	miR-6089 (2)	2,073 ÷ 2,078	−132 ÷−136	89 ÷ 91	24
	ID01382.3p-miR	2,075	−113	93	20
	miR-3620-5p (2)	2,069 ÷ 2,074	−117 ÷−115	87 ÷ 89	22
SOX4**	ID01839.3p-miR	2,994	−123	89	23
13.2	ID01282.3p-miR	3,000	−125	95	23
	ID03445.3p-miR	3,000	−127	90	24
	ID00101.3p-miR	3,001	−115	92	22
TGFB1**	ID03306.3p-miR	2,060	−123	94	21
19.5	miR-6089 (4)	2,060 ÷ 2,095	−132 ÷−136	89 ÷ 91	24
	ID01382.3p-miR	2,062	−113	93	20
	miR-3620-5p	2,086	−115	87	22
	ID03208.5p-miR	2,066	−125	88	24
	ID00978.5p-miR	2,089	−119	90	22
	ID00296.3p-miR	2,093	−140	89	25

**Notes.**

Genes without *—miRNA binding sites are in the 5′UTR, genes with *—miRNA binding sites are in the CDS, **—miRNA binding sites are in the 3′UTR; values in parentheses indicate the number of binding sites; ÷—the change of the parameter in the interval. Different repeating clusters of miRNA binding sites in different genes are coloured.

The formation of a cluster of BSs for the *FOXA1* gene in 5′UTR indicated this gene’s capability for compaction, which causes competition among miRNA for the BS. Despite the fact that ID01099.5p-miR, ID01190.5p-miR and ID02457.3p-miR are fully complementary to the mRNA gene, they had a free energy interaction of −108 kJ/mole, which is significantly less than that of the other miRNAs. At equal concentrations of all miRNAs, ID00252.5p-miR, ID00296.3p-miR and ID01702.3p-miR had ΔG values of −140 kJ/mole because of their advantage in binding to the mRNA of the *FOXA1* gene. The average free energy of miRNA binding, without the three miRNAs with a length of 17 nt, was −126 kJ/mole, which is characteristic of miRNA binding in 5′UTR.

BSs of 15 miRNAs were in 5′UTR of the *HMGA2* mRNA cluster from 529 nt to 607 nt. The total length of BSs was 4.6 times greater than the cluster length. ID00296.3p-miR and ID00296.3p-miR had ΔG values of −142 kJ/mole and −146 kJ/mole, respectively.

The *ITGB1* gene had no 5′UTR, but a cluster for five miRNA BSs was located from 91 nt to 121 nt in the beginning of CDS with a length of 31 nt, which is 3.5 times less than the sum of the lengths of five miRNAs.

For the *HMGA2* gene, there was a cluster with a length of 41 nt for four BSs, with the BS total length of 88 nt.

Apparently, BSs compaction is associated not only with a decrease in the length of the gene, but also with increased competition between miRNAs. Take for example, the cluster of eight BSs with 3′UTR of the mRNA *SMAD3* gene with a length of 35 nt. Only one miRNA can be bound in a cluster. At equal concentrations of all six miRNAs, ID02822.5p-miR and miR-6089, with free interaction energy of −127 kJ/mole to −136 kJ/mole, would have an advantage in binding to the cluster.

The 3′UTR of the mRNA *SOX4* gene had four miRNA BSs organized in a cluster of 29 nt. ID01282.3p-miR and ID03445.3p-miR bound to mRNA had ΔG values of −125 kJ/mole and −127 kJ/mole, respectively.

The mRNAs of the *TGFB1* gene had a BS cluster for seven miRNAs with a length of 60 nt. The length of 3′UTR was 146 nt with 10 miRNA BSs equal to 230 nt, compacting the BS 4.8 times.

Figure S2 shows the schemes of interaction among some miRNAs with mRNA of several candidate genes of the luminal A and B subtypes. The presented schemes clearly show the advantage of the MirTarget program in predicting the miRNA BSs. For example, ID03367.5p-miR formed a non-canonical G-U pair in the mRNA *FOXA1* gene. However, ID03367.5p-miR was able to bind to 19 nucleotides of mRNA and the free energy interaction was 93% of the maximum value. The ID02542.5p-miR interacted with 23 nucleotides of the mRNA *FOXA1* gene, but had only one unpaired nucleotide. Similar interactions between the miRNAs and their target genes are valid for the following pairs: ID00101.3p-miR and the *HMGA2* gene, ID00849.3p-miR and the *HMGA2* gene, miR-4507-3p and the *SMAD3* gene, miR-937-5p and the *TGFB1* gene, miR-937-5p and the *SMAD3* gene, and ID01403.5p-miR and the *HMGA2* gene.

### Subtype HER2 breast cancer

Fifteen miRNAs were bound in 5′UTR mRNAs of three candidate genes of the breast cancer subtype HER2 (Table 3). The mRNA of the *EPOR* gene had three miRNA BSs, with overlapping nucleotide sequences. Three BSs of ID01633.3p-miR, ID01599.3p-miR and ID01626.3p-miR comprised a 26 nt cluster located in 5′UTR of the mRNA *EPOR* gene. Without overlapping sites, the length of three miRNAs would be 67 nt, which is half of the 135 nt length of 5′UTR. Consequently, the compacting of miRNA BSs is useful in reducing the proportion of BSs by 2.6 times in 5′UTR of the mRNA *EPOR* gene.

In the mRNA of the *MAZ* gene, the BSs of ID00968.3p-miR, ID01476.3p-miR, miR-1470, and ID00620.3p-miR were located in a cluster with a length of 34 nt. The total length of the four miRNAs was equal to 87 nt. Another cluster in *MAZ* mRNA with a length of 44 nt was formed by miR-6850-5p, miR-4466, miR-762, ID00915.3p-miR and ID02979.5p-miR BSs. Both clusters occupied only 78 nt, and the total length of BSs of nine miRNAs was 196 nt.

In the mRNA of the *NISCH* gene, the BSs of ID03445.3p-miR, ID01560.3p-miR and ID03119.5p-miR formed a cluster with a length of 35 nt. With cluster formation, the length of these BSs was 71 nt, i.e., 52% of the length of 5′UTR.

**Table 3:** Characteristics of miRNA interaction in mRNA genes of BC subtype HER2.

Gene, RPKM	miRNA	Start of binding site, nt	ΔG, kJ/mole	ΔG/ΔGm, %	Length, nt
*EPOR*	ID01633.3p-miR	77	−108	91	21
8.1	ID01599.3p-miR	79	−119	89	23
	ID01626.3p-miR	80	−129	90	23
*MAZ*	ID00968.3p-miR	16	−117	93	20
9.5	ID01476.3p-miR	16	−134	91	23
	miR-1470	18	−123	97	21
	ID00620.3p-miR	27	−127	91	23
	miR-6850-5p	92	−115	87	22
	miR-4466	107	−110	98	18
	miR-762	111	−123	91	22
	D00915.3p-miR	112	−127	88	24
	ID02979.5p-miR	114	−121	92	22
*NISCH*	ID03445.3p-miR	31	−125	88	24
32.2	ID01560.3p-miR	38	−123	89	23
	ID03119.5p-miR	41	−125	88	24
MAPK3*	ID00149.3p-miR	1,144	−117	93	22
32.6	ID01748.3p-miR	1,144	−110	91	21
	miR-6805-3p	1,145	−117	87	23
MAZ*	miR-6729-5p	361	−115	87	22
9.5	ID02623.3p-miR	363	−125	89	23
	ID02460.5p-miR	372	−119	92	22
	miR-2861	375	−110	95	19
	ID02294.5p-miR (3)	457 ÷ 469	−134 ÷−138	91 ÷ 94	24
	ID02986.5p-miR	459	−119	93	21
	ID01819.5p-miR	461	−125	87	25
	ID01804.3p-miR (2)	464 ÷ 467	−140	88	25
	ID02064.5p-miR	489	−121	92	21
	ID02538.3p-miR	489	−125	94	22
	ID00296.3p-miR	500	−138	88	25
	ID01641.3p-miR	506	−132	89	24
	miR-3960	505	−119	95	20
	miR-4706	605	−123	87	25
	ID01705.3p-miR	608	−117	92	21
	ID01641.3p-miR	608	−134	90	24
	miR-3960	612	−117	93	20
	ID01768.3p-miR	893	−113	90	22
	ID01911.5p-miR	900	−123	89	23
	ID00849.3p-miR	901	−125	97	22
BRCA2**	ID00112.5p-miR	10,722	−102	91	21
0.1	ID02744.3p-miR	10,738	−104	92	22
	miR-619-5p	10,746	−117	96	22
CDK6**	miR-548h-3p	1,677	−104	91	23
2.2	miR-548z	1,677	−104	91	23
	miR-548aq-3p	1,678	−102	94	22
	miR-548az-3p	1,678	−98	94	21
	ID03264.3p-miR	1,678	−98	90	22
	miR-466 (10)	1,908 ÷ 1,926	−104 ÷−108	90 ÷ 93	23
	ID01030.3p-miR (7)	1,900 ÷ 1,918	−108 ÷−115	89 ÷ 95	23
	ID00436.3p-miR (9)	1,896 ÷ 1,920	−104 ÷−106	89 ÷ 91	23
	ID02513.5p-miR	1,901	−102	91	22

**Notes.**

Genes without *—miRNA binding sites are in the 5′UTR, genes with *—miRNA binding sites are in the CDS, **—miRNA binding sites are in the 3′UTR; values in parentheses indicate the number of binding sites; ÷—the change of the parameter in the interval. Different repeating clusters of miRNA binding sites in different genes are coloured.

**Figure 3:**
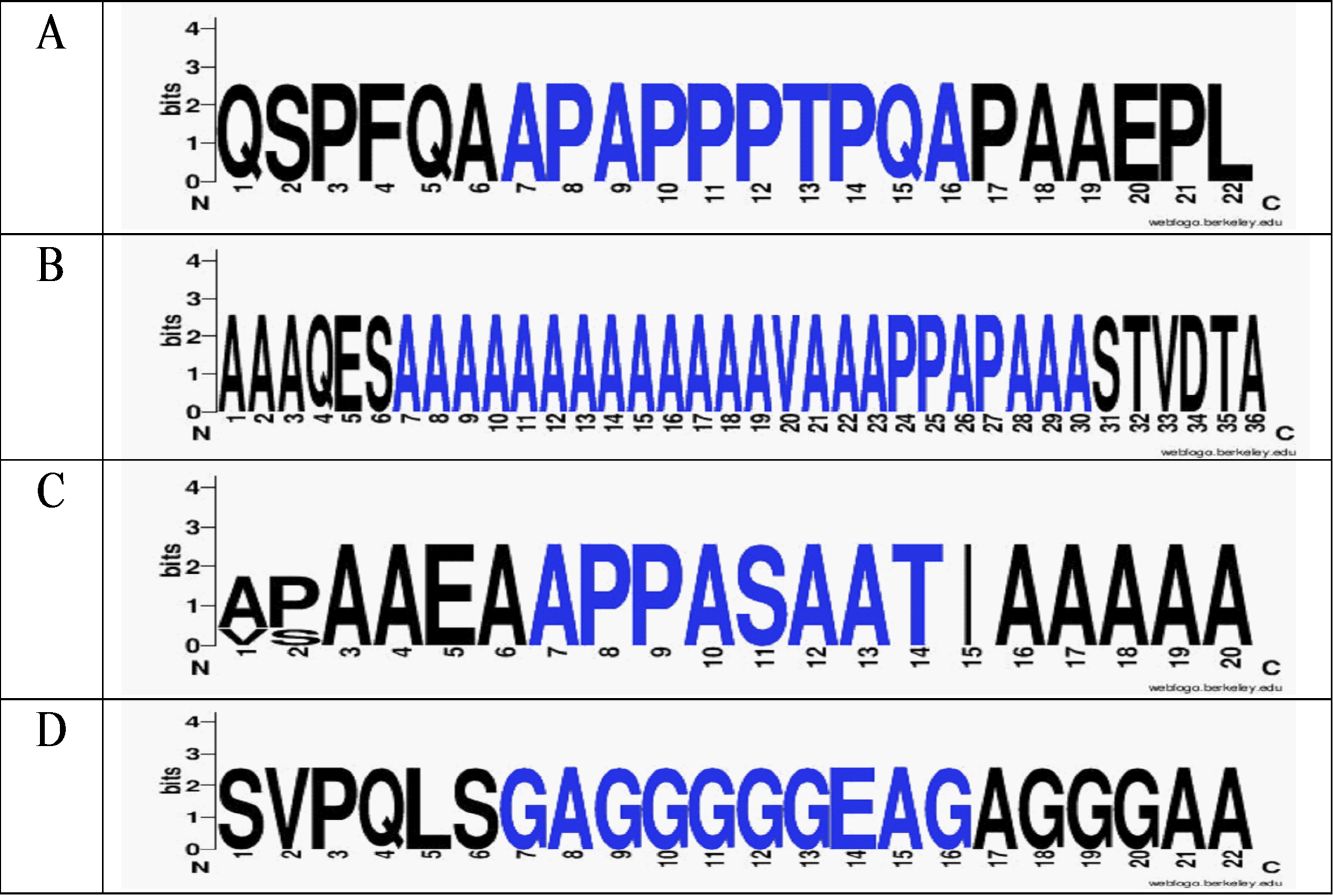
Logo plots of variation of amino acids in the region of orthologous MAZ proteins. Orthologous MAZ proteins containing: APAPPPTPQA oligopeptide (A), AAAAAAAAAAAAAVAAAPPAPAAA oligopeptide (B), APPASAAT oligopeptide (C), and GAGGGGGEAG oligopeptide (D) of *Hsa, Pab, Ptr, Csa*. Conservative oligopeptides are highlighted in blue.

There were 24 miRNAs for which the mRNA was targeted in CDS. *MAPK3* gene was a target of three miRNAs, the BSs of which were located in a cluster with a length of 26 nt. The mRNA of the *MAZ* gene had miRNA BSs with nucleotide sequences overlapping into four different clusters (Table 3). The first cluster with a length of 33 nt included BSs of miR-6729-5p, ID02623.3p-miR, ID02460.5p-miR and miR-2861. Nucleotide sequences of BS clusters encoded the oligopeptide APAPPPTPQA, which was conservative in orthologous proteins MAZ of *H. sapiens, P. abelii, P. troglodytes,* and *C. sabaesus* (Fig. 3A). The second cluster was located from 457 nt to 530 nt. The total length of all BSs of miRNAs of this cluster was 302 nt. This length required BS compaction since all nucleotides participate in the coding of functionally important amino acids in CDS. Despite the long length of the cluster of nine miRNA BSs encoding the oligopeptide AAAAAAAAAAAAAVAAAPPAPAAA, the MAZ orthologs were conservative (Fig. 3B). The third cluster consisted of miR-4706, ID01641.3p-miR, ID01705.3p-miR, and miR-3960 BSs with a length of 30 nt. The cluster encoded the conservative oligopeptide APPASAAT (Fig. 3C). The fourth cluster, with a length of 30 nt, included BSs for three miRNAs. The encoded subcluster oligopeptide GAGGGGGEAG was also conservative (Fig. 3D).

All BSs for miRNAs that interacted with *MAZ* mRNA had a total length of 472 nt, which was approximately 33% of the total CDS length. Clustered BSs for miRNA occupied only 1,434 nt, 12% of CDS length. The *MAZ* gene was the most preferable target for miRNA, so monitoring its expression should be a priority.

Fifteen miRNAs were bound within the mRNA of candidate breast cancer subtype HER2 genes with a free energy of −125 kJ/mole or greater (Table 3). For example, ID01626.3p-miR had a competitive advantage over ID01633.3p-miR and ID01599.3p-miR for binding in the mRNA cluster *EPOR*. In two clusters of *MAZ* mRNA, ID01476.3p-miR and ID00915.3p-miR were predominantly bound. The translation of the mRNA *MAZ* gene will be significantly suppressed if ID02294.5p-miR (which has three BSs) is present, and ID01804.3p-miR and ID00296.3p-miR have sites with a free binding energy of −140 kJ/mole and −138 kJ/mole, respectively.

In 3′UTR of the mRNA *BRCA2* gene, three miRNA BSs were identified with overlapping nucleotide sequences (Table 3). The *CDK6* gene was a target for nine miRNAs. miR-548h-3p, miR-548z, miR-548aq-3p, miR-548az-3p, and ID03264.3p-miR formed a cluster from 1,677 nt to 1,699 nt. The mRNA of *BRCA2* and *CDK6* genes had BSs for miRNA in 3′UTR with a low free energy of binding: −98 kJ/mole to −117 kJ/mole. In the 3′UTR of the mRNA *CDK6* gene with an RPKM value of 2.2, there were 10 BSs of miR-466, nine BSs of ID00436.3p-miR, and seven BSs of ID01030.3p-miR forming a cluster from 1,896 nt to 1,948 nt (Table 3). Multiple BSs for these miRNAs allowed them to bind with mRNA and significantly increased the probability of translation inhibition of the mRNA *CDK6* gene.

Compacting miRNA BSs is difficult to explain if its sole purpose is conserving the length of 3′UTR, since there are various reasons for compacting BSs. For example, the binding of one miRNA precludes other miRNAs binding with that site. If this miRNA is a signal of the host gene (gene encoding miRNA), the target gene will not perceive this signal. Specifically, there is competition between different miRNAs for the BS, and for the ability to regulate the expression of the target gene.

It should be noted that most miRNA BSs were located at the beginning of 5′UTR and CDS mRNA regions of the *MAZ* gene (Table 3). This localization of miRNA BSs allows protein synthesis to be ended earlier in the case of the formation of abortive proteins. For example, the first three clusters of miRNA BSs were located in CDS of the mRNA *MAZ* gene. All BSs of nine miRNAs in 5′UTR of mRNA *MAZ* were located between 16 nt and 114 nt of the 168 nt length of 5′UTR.

The miRNA BSs in 3′UTR of mRNA *BRCA2* and *CDK6* genes were also located at the beginning of 3′UTR (Table 3).

Figure S3 shows the schemes of interaction of miRNAs with mRNAs of candidate genes of the subtype HER2. Several miRNAs and their target genes (ID02998.3p-miR and *MAZ* gene, miR-5008-5p and *MAZ* gene, ID02499.3p-miR and *MAZ* gene, miR-6805-3p and *MAPK3* gene, miR-3960 and *MAZ* gene, miR-877-3p, miR-7111-3p, and ID01352.3p-miR) had BSs in the same region of the mRNA *MAZ* gene from 2,273 nt to 2,774 nt. All miRNA nucleotides formed hydrogen bonds with this region of mRNA.

After the discovery of clusters of miRNA BSs with mRNA of candidate genes of breast cancer subtypes, the question arises as to how stable these structural forms are. It is known that some miRNAs arose in early stages of evolution and have been stable for tens of millions of years of species divergence (Kondybayeva et al., 2018). Other miRNA associations with mRNA have appeared recently, and they have not been observed even in closely related species. Taking this into consideration, we checked the variability of nucleotide sequences of BSs in the clusters we identified. The analysis results of nucleotide sequences of clusters in mRNA candidate breast cancer subtypes are given in Tables S3–S5. The data obtained show that in most cases the nucleotide sequences of clusters are identical. Observed differences in single nucleotides show slight changes in the degree of interaction between miRNA and BSs. Consequently, established bindings between miRNAs and BSs organized in clusters are stable in genomes of studied objects which have diverged over millions of years. Evolutionary conservatism allows options among adequate animal models for studying associations between miRNAs and mRNAs.

## Discussion

Dysregulation of gene expression by miRNAs is one of the causes of oncogenesis (Adhami et al., 2018), and studies about the participation of miRNAs in oncogenesis testifies to their important role in this process (Persson et al., 2011). Many studies have been devoted to the role of miRNA in various diseases, including triple-negative BC (Bar et al., 2017; Buschmann et al., 2018; Yao et al., 2018), luminal A and B subtypes of BC (Aure et al., 2017; Hannafon et al., 2016; Wang & Luo, 2015) and the HER2 subtype of BC (Halvorsen et al., 2017; Patel et al., 2016; Wang & Lin, 2013). However, there have been few reliably established associations between miRNA and target genes. In this article, we identified associations of the miRNAs with their target genes that may be responsible for the development of breast cancer, taking into account its subtypes. The selectivity of miRNA and mRNA interaction, as well as the facts of preservation of the entire miRNA nucleotide sequence and the corresponding BSs in the mRNA of target genes, demonstrate the stability of these interactions over tens of millions of years of evolution (Yurikova et al., 2019). We applied new bioinformatics approaches to the assessment of these relationships, which allowed us to reveal important characteristics of the binding between miRNAs and target genes (Tables 1–3). Previously experimentally established miRNA BSs using the mRNA of target *RTL1* genes (Davis et al., 2005) were verified using the MirTarget program. The *RTL1* gene is located on the antisense sequence for 10 miRNA, and participates in oncogenesis through them. For example, miR-127-5p can suppress the expression of a tumor suppressor gene (Wang et al., 2011a), miR-136-5p can regulate the expression of *CLDN15* (Yurikova et al., 2019)*, ENAH* genes are involved in tumor invasion (Forse et al., 2015; Takehara et al., 2009), and miR-432-3p can affect the gene *IL2RB* involved in the development of breast cancer (García-Tuñón et al., 2004). With the MirTarget program, we confirmed the seven experimentally established BSs for miR-127, miR-136, miR-431, miR-433-3p/5p, and miR-434-3p/5p with the mRNA of the *RTL1* gene, and we predicted another three BSs for miRNAs (Davis et al., 2005). All 10 miRNA BSs were located in the CDS of the mRNA gene, and the nucleotide sequences of miRNA and mRNA were fully complementary. Note that the nucleotide sequences miR-127-5p and miR-127-3p were conservative in species that had diverted tens of millions of years ago, according to miRBase (Yurikova et al., 2019). Using the MirTarget program, we predicted miRNA BSs in the CDS, 5′UTR and 3′UTR of many genes (Atambayeva et al., 2017; Ivashchenko et al., 2016; Ivashchenko et al., 2014a; Ivashchenko et al., 2014b; Ivashchenko, Issabekova & Berillo, 2013). The nucleotide sequences of miRNA and their BSs have been conserved in the mRNA genes of animal and plant organisms over tens of millions of years of evolution (Bari, Orazova & Ivashchenko, 2013; Ivashchenko et al., 2016; Ivashchenko et al., 2014a; Ivashchenko et al., 2014b; Ivashchenko, Issabekova & Berillo, 2013).

The MirTarget program detects BS organization with overlapping nucleotide sequences. The organization of BSs into clusters has two consequences: (a) BSs are compacted to reduce their share in the total length of mRNA; (b) competition is created among miRNAs to bind to mRNA, taking into account the free energy of miRNA interaction with mRNA.

The competition between miRNA complicated our interpretation of the expected effects of changing the miRNA concentration. In diseases, the concentration of miRNA can increase and decrease dozens of times (Lu et al., 2016; Yang, Sui & Liang, 2017). For example, a decrease in the concentration of one miRNA may not cause any effect, since other miRNAs will inhibit protein translation. In most cases, the binding of miRNA with mRNA is unlikely to lead to a complete suppression of translation, such as with a fully complementary interaction of miRNA with mRNA (Davis et al., 2005). Even a few miRNAs will not cause a complete suppression of translation if their concentration is less than the concentration of mRNA.

It has been found that some miRNAs may bind to the mRNA of more than one candidate gene of different subtypes. For example, ID03445.3p-miR may interact with the mRNA of the *NISCH* gene of the HER2 subtype, mRNA of the *RAB5A* gene of the triple-negative subtype, and mRNA of the *SOX4* gene of luminal A and B subtypes (Tables 1–3). Additionally, ID00061.3p-miR can bind to mRNA of the *RAB5A* gene of the triple-negative subtype and mRNA of the *FOXA1* gene of luminal A and B subtypes (Tables 1 and 2). ID01641.3p-miR can interact with mRNA of *FOXA1, HMGA2* genes of luminal A and B subtypes, and with mRNA of the *MAZ* gene of the HER2 subtype (Tables 2 and 3). miR-466 could bind to mRNA of *RUNX1, SFN* and *CDK6* genes (Tables 1 and 3). One miRNA can interact with mRNA of two or more candidate genes of the same subtype. For example, miR-6089 can bind to mRNA of *SMAD3* and *TGFB1* genes when it has two and four consecutive sites (Table 2), and to mRNA of the *SFN* gene with one BS (Table 1). The ID00367.5p-miR and miR-1273g-3p BSs were located through seven nucleotides in mRNA of the *ATM* gene and *STMN1* gene (Table 1). BSs of these miRNAs were part of corresponding clusters.

A distinctive feature of candidate genes of the triple-negative subtype is the presence of several genes of miR-1273 family BSs in mRNA (Table 1). The mRNA of the *IL11* gene in the cluster included BSs of miR-1273d, miR-1273e and miR-1273f. The mRNA genes of *ATM, CBL,* and *STMN1* included BSs of miR-1273a and miR-1273g-3p, with a distance of 22 nt between the origins of their BSs. Characteristics of the interactions of the miR-1273 family with candidate genes of the triple-negative subtype are not random. mRNA of candidate genes of luminal A and B, and HER2 subtypes lack the BSs of the miR-1273 family (Tables 2 and 3). We predicted several multiple BSs for a single miRNA. For example, miR-466 had two BSs in mRNA of the *RUNX1* gene, six BSs in mRNA of the *SFN* gene, and 10 BSs in mRNA of the *CDK6* gene (Tables 1 and 3). BSs of ID01030.3p-miR and ID00436.3p-miR were included in clusters of mRNA BSs of *ATM* and *SFN* genes (Table 1). The mRNA of the *SFN* gene had six BSs with ID01030.3p-miR and ID00436.3p-miR. It is known that single nucleotide, dinucleotide, and trinucleotide repeats are found in mRNA genes, and which nucleotide repeats are targets for miRNA have been shown (Kondybayeva et al., 2018; Niyazova et al., 2015). The position of BSs in the same cluster suggests competition between miRNA to bind to mRNA of the target gene. Competition among miRNAs for BSs also occurs when they are bound to mRNA of different genes if they are expressed both in a single cell and in different cells of the body, since miRNA is transported through the body with blood (Hannafon et al., 2016; Lagendijk et al., 2018; Zhang et al., 2017). It is necessary to take into account the concentration of miRNA and mRNA to explain the effectiveness of their interaction.

Some miRNAs can bind with high free energy to mRNA of targets genes of different subtypes: ID.01804.3p-miR and *RAB5A*, *MAZ* (−140 kJ/mole); ID.00252.5p-miR and *FOXA1* (−140 kJ/mole); ID.00296.3p-miR and *FOXA1*, *HMGA2* (−146 kJ/mole); and ID.01641.3p-miR and *HMGA2* (−142 kJ/mole) (Tables 1–3). Such associations of miRNA and target genes can be used as markers of two BC subtypes, since the expression of these genes will be significantly suppressed by the corresponding miRNA. Simultaneously while controlling the expression of these miRNA and gene associations, it is necessary to control the expression of specific associations for each subtype. For example, such associations may be for ID.03332.3p-miR, ID.02761.3p-miR and the *CBL* gene; ID.02930.3p-miR, ID.01804.3p-miR and the *RAB5A* gene for triple-negative subtype (Table 1); ID.01702.3p-miR and the *FOXA1* gene for the luminal subtype A and B (Table 2); or ID.1476.3p-miR, ID.02294.5p-miR and the *MAZ* gene for the subtype HER2 (Table 3).

Tables 1–3 provide information (RPKM) on the normal expression of candidate genes in the mammary gland. The most highly expressed genes are *MMP2* (Table 1), *ITGB1* (Table 2), *NISCH,* and *MAPK3* (Table 3). The mRNA of *MMP2* and *ITGB1* genes contain clusters of BSs for five miRNAs, and the mRNA of *NISCH* and *MAPK3* genes contain clusters for three miRNAs. Consequently, the expression of these candidate genes and miRNAs binding in respective clusters can be used to develop methods for diagnosing BC subtypes. The *HMGA2* gene is not normally expressed (Table 2), but its mRNA has two binding clusters for 18 miRNAs, and some miRNAs can bind with high free energy to mRNA, suggesting the suppression of its possible expression. Several studies have shown that this gene can be expressed in tumor cells and its increased expression leads to the development of oncogenesis (Chen et al., 2019; Niu et al., 2019; Pearlman et al., 2019; Piscuoglio et al., 2012; Sun et al., 2014; Wang et al., 2011b).

For each of the BC subtypes, two or more miRNAs with binding sites in the same cluster and in several genes were detected; for example, identified BS clusters for miR-1273a and miR-1273g-3p in *ATM, CBL* and *STMN1* genes of the triple-negative subtype (Table 1). For miR-466, ID.01030.3p-miR and ID.00436.3p-miR binding sites were found in clusters mRNA of *RUNX1* and *SFN* genes of the triple-negative subtype. These two associations of miRNAs and candidate genes can serve as markers for the diagnosis of a triple-negative subtype. For miR-466, ID.01030.3p-miR and ID.00436.3p-miR binding sites were found in the cluster mRNA of *CDK6* candidate gene of the HER2 subtype. Consequently, in addition to controlling the concentration of these three miRNAs, the control of the expression of target genes is necessary to establish the disease subtype.

The mRNA *SMAD3* and *TGFB1* genes of luminal A and B subtypes contain clusters of binding sites of miR-6089, ID.01382.3p-miR and miR-3620-5p, which justify the use of these associations between miRNAs and genes as markers of luminal A and B subtypes. However, the candidate *SFN* gene of the triple-negative subtype also contains an miR-6089 binding site. Therefore, in this case it is also necessary to control the expression of *SFN* gene to identify the disease subtype. The examples above show that for accurate labeling of disease subtypes, it is imperative to control the expression of miRNAs and target genes. The same examples show the inadequacy of diagnosing using one or several miRNAs with concentrations changing correlatively across different subtypes without controlling the influence of these miRNAs on target genes. The results of this work show the necessity for simultaneous quantitative control of the expression of many genes and miRNAs.

Our proposed use of the associations between miRNA and target genes should be analyzed, while taking into account the following factors: (a) miRNA and their target genes perform limited stages of key biological processes involved in the development of diseases; (b) these interactions between miRNA with mRNA have high free energy; (c) there is a greater number of miRNAs that bind to mRNA; and (d) included miRNAs have more target genes. Depending on circumstances, the adequacy and significance of the associations listed between miRNAs and mRNA may vary.

## Conclusions

Using the associations between miRNAs and their targets genes has been identified as a method for identifying breast cancer subtypes. The clustering of miRNA BSs decreases the fraction of BSs with nucleotide sequences in mRNA. The cluster organization of miRNA BSs is mainly found in 5′UTR and 3′UTR. In the CDS, the share of miRNA BSs organized into clusters is less than that of single miRNA BSs. The cluster organization of miRNA BSs, together with the free energy of miRNA interaction with mRNA, causes competition between miRNAs to bind to mRNA. This phenomenon demonstrates the competitive relationship among miRNAs in the regulation of the expression of target genes. The number of miRNA BSs in clusters indicates the degree of dependence of the expression of target genes on the expression of other miRNA host genes. Our results show the necessity for simultaneous quantitative control of the expression of many genes and many miRNAs in the development of methods to diagnose studied breast cancer subtypes.

##  Supplemental Information

10.7717/peerj.8049/supp-1 Figure S1 Schemes of miRNA interaction with mRNA of candidate genes of breast cancer triple-negative subtype

10.7717/peerj.8049/supp-2 Figure S2 Schemes of miRNA interaction with mRNA of candidate genes of breast cancer luminal A and B subtypes

10.7717/peerj.8049/supp-3 Figure S3 Schemes of miRNA interaction with mRNA of candidate genes of breast cancer HER2 subtype

10.7717/peerj.8049/supp-4 Table S1 Candidate genes of subtypes of breast cancer, indicating sources of information on their participation in oncogenesis of breast cancer

10.7717/peerj.8049/supp-5 Table S2 Information on miRNAs interacted with genes of breast cancer and other cancer

10.7717/peerj.8049/supp-6 Table S3 The nucleotide sequence of clusters of miRNA binding sites in orthologous candidate genes of triple-negative subtype

10.7717/peerj.8049/supp-7 Table S4 The nucleotide sequence of clusters of miRNA binding sites in orthologous candidate genes of luminal A and B subtypes

10.7717/peerj.8049/supp-8 Table S5 The nucleotide sequence of clusters of miRNA binding sites in orthologous candidate genes of HER2 subtype

## Additional Information and Declarations

### Data Availability

The following information was supplied regarding data availability: The nucleotide sequences of candidate genes (ID) of BC subtypes are available at NCBI. The accession numbers are in Table S1. The nucleotide sequences of 2565 miRNAs from miRBase and 3707 miRNAs are available from: Novel primate- and tissue-specific microRNAs, Proceedings of the National Academy of Sciences Mar 2015, 112 (10) E1106–E1115; DOI: 10.1073/pnas.1420955112
Protein expression was found by the corresponding gene (ID) in the Human Protein Atlas. Reads Per Kilobase Million (RPKM) value is available in the Human Protein Atlas data (https://www.proteinatlas.org/ENSG00000150093-ITGB1/tissue/breast).

## References

[ref-1] Adhami M, Haghdoost AA, Sadeghi B, Malekpour AR (2018). Candidate miRNAs in human breast cancer biomarkers: a systematic review. Breast Cancer.

[ref-2] Atambayeva S, Niyazova R, Ivashchenko A, Pyrkova A, Pinsky I, Akimniyazova A, Labeit S (2017). The binding sites of miR-619-5p in the mRNAs of human and orthologous genes. BMC Genomics.

[ref-3] Aure MR, Vitelli V, Jernström S, Kumar S, Krohn M, Due EU, Haukaas TH, Leivonen SK, Vollan HK, Lüders T, Rødland E, Vaske CJ, Zhao W, Møller EK, Nord S, Giskeødegård GF, Bathen TF, Caldas C, Tramm T, Alsner J, Overgaard J, Geisler J, Bukholm IR, Naume B, Schlichting E, Sauer T, Mills GB, Kåresen R, Mælandsmo GM, Lingjærde OC, Frigessi A, Kristensen VN, Børresen-Dale AL, Sahlberg KK (2017). Integrative clustering reveals a novel split in the luminal A subtype of breast cancer with impact on outcome. Breast Cancer Research.

[ref-4] Bar I, Merhi A, Abdel-Sater F, Ben Addi A, Sollennita S, Canon JL, Delrée P (2017). The MicroRNA miR-210 is expressed by cancer cells but also by the tumor microenvironment in triple-negative breast cancer. Journal of Histochemistry and Cytochemistry.

[ref-5] Bari A, Orazova S, Ivashchenko A (2013). miR156- and miR171-binding sites in the protein-coding sequences of several plant genes. BioMed Research International.

[ref-6] Benson JR, Jatoi I (2012). The global breast cancer burden. Future Oncology.

[ref-7] Biagioni F, Bossel BMN, Fontemaggi G, Canu V, Mori F, Antoniani B, Di Benedetto A, Santoro R, Germoni S, De Angelis F, Cambria A, Avraham R, Grasso G, Strano S, Muti P, Mottolese M, Yarden Y, Domany E, Blandino G (2012). miR-10b*, a master inhibitor of the cell cycle, is down-regulated in human breast tumours. EMBO Molecular Medicine.

[ref-8] Blenkiron C, Goldstein LD, Thorne NP, Spiteri I, Chin SF, Dunning MJ, Barbosa-Morais NL, Teschendorff AE, Green AR, Ellis IO, Tavaré S, Caldas C, Miska EA (2007). MicroRNA expression profiling of human breast cancer identifies new markers of tumor subtype. Genome Biology.

[ref-9] Buschmann D, González R, Kirchner B, Mazzone C, Pfaffl MW, Schelling G, Steinlein O, Reithmair M (2018). Glucocorticoid receptor overexpression slightly shifts microRNA expression patterns in triple-negative breast cancer. International Journal of Oncology.

[ref-10] Chen X, Zeng K, Xu M, Liu X, Hu X, Xu T, He B, Pan Y, Sun H, Wang S (2019). P53-induced miR-1249 inhibits tumor growth, metastasis, and angiogenesis by targeting VEGFA and HMGA2. Cell Death & Disease.

[ref-11] Davis E, Caiment F, Tordoir X, Cavaillé J, Ferguson-Smith A, Cockett N, Georges M, Charlier C (2005). RNAi-mediated allelic trans-interaction at the imprinted Rtl1/Peg11 locus. Current Biology.

[ref-12] Enerly E, Steinfeld I, Kleivi K, Leivonen SK, Aure MR, Russnes HG, Rønneberg JA, Johnsen H, Navon R, Rødland E, Mäkelä R, Naume B, Perälä M, Kallioniemi O, Kristensen VN, Yakhini Z, Børresen-Dale AL (2011). miRNA-mRNA integrated analysis reveals roles for miRNAs in primary breast tumors. PLOS ONE.

[ref-13] Forse CL, Agarwal S, Pinnaduwage D, Gertler F, Condeelis JS, Lin J, Xue X, Johung K, Mulligan AM, Rohan TE, Bull SB, Andrulis IL (2015). Menacalc, a quantitative method of metastasis assessment, as a prognostic marker for axillary node-negative breast cancer. BMC Cancer.

[ref-14] García-Tuñón I, Ricote M, Ruiz A, Fraile B, Paniagua R, Royuela M (2004). Interleukin-2 and its receptor complex (alpha, beta and gamma chains) in *in situ* and infiltrative human breast cancer: an immunohistochemical comparative study. Breast Cancer Research.

[ref-15] Halvorsen AR, Helland A, Gromov P, Wielenga VT, Talman MM, Brunner N, Sandhu V, Børresen-Dale AL, Gromova I, Haakensen VD (2017). Profiling of microRNAs in tumor interstitial fluid of breast tumors—a novel resource to identify biomarkers for prognostic classification and detection of cancer. Molecular Oncology.

[ref-16] Hannafon BN, Trigoso YD, Calloway CL, Zhao YD, Lum DH, Welm AL, Zhao ZJ, Blick KE, Dooley WC, Ding WQ (2016). Plasma exosome microRNAs are indicative of breast cancer. Breast Cancer Research.

[ref-17] Huggett J, O’Grady J (2014). Molecular diagnostics: current research and applications.

[ref-18] Ivashchenko A, Berillo O, Pyrkova A, Niyazova R (2014b). Binding sites of miR-1273 family on the mRNA of target genes. Biomed Research International.

[ref-19] Ivashchenko A, Berillo O, Pyrkova A, Niyazova R, Sh Atambayeva (2014a). The properties of binding sites of miR-619-5p, miR-5095, miR-5096 and miR-5585-3p in the mRNAs of human genes. Biomed Research International.

[ref-20] Ivashchenko A, Issabekova AS, Berillo OA (2013). miR-1279, miR-548j, miR-548m, and miR-548d-5p binding sites in CDSs of paralogous and orthologous PTPN12, MSH6, and ZEB1 Genes. BioMed Research International.

[ref-21] Ivashchenko AT, Pyrkova AY, Niyazova RY, Alybayeva A, Baskakov K (2016). Prediction of miRNA binding sites in mRNA. Bioinformation.

[ref-22] Jemal A, Center MM, DeSantis C, Ward EM (2010). Global patterns of cancer incidence and mortality rates and trends. Cancer Epidemiology, Biomarkers & Prevention.

[ref-23] Kondybayeva AM, Akimniyazova AN, Kamenova SU, Ivashchenko AT (2018). The characteristics of miRNA binding sites in mRNA of ZFHX3 gene and its orthologs. Vavilov Journal of Genetics and Breeding.

[ref-24] Kool ET (2001). Hydrogen bonding, base stacking, and steric effects in DNA replication. Annual Review of Biophysics and Biomolecular Structure.

[ref-25] Kurozumi S, Yamaguchi Y, Kurosumi M, Ohira M, Matsumoto H, Horiguchi J (2017). In microRNA research into breast cancer with particular focus on the associations between microRNAs and intrinsic subtypes. Journal of Human Genetics.

[ref-26] Lagendijk M, Sadaatmand S, Koppert LB, Tilanus-Linthorst MMA, De Weerd V, Ramírez-Moreno R, Smid M, Sieuwerts AM, Martens JWM (2018). MicroRNA expression in pre-treatment plasma of patients with benign breast diseases and breast cancer. Oncotarget.

[ref-27] Lee CH, Kuo WH, Lin CC, Oyang YJ, Huang HC, Juan HF (2013). MicroRNA-regulated protein-protein interaction networks and their functions inbreast cancer. International Journal of Molecular Sciences.

[ref-28] Lemieux S, Major F (2002). RNA canonical and non-canonical base pairing types: a recognition method and complete repertoire. Nucleic Acids Research.

[ref-29] Leontis NB, Stombaugh J, Westhof E (2002). The non-Watson-Crick base pairs and their associated isostericity matrices. Nucleic Acids Research.

[ref-30] Londin E, Loher P, Telonis AG, Quann K, Clark P, Jing Y, Hatzimichael E, Kirino Y, Honda S, Lally M, Ramratnam B, Comstock CE, Knudsen KE, Gomella L, Spaeth GL, Hark L, Katz LJ, Witkiewicz A, Rostami A, Jimenez SA, Hollingsworth MA, Yeh JJ, Shaw CA, McKenzie SE, Bray P, Nelson PT, Zupo S, Van Roosbroeck K, Keating MJ, Calin GA, Yeo C, Jimbo M, Cozzitorto J, Brody JR, Delgrosso K, Mattick JS, Fortina P, Rigoutsos I (2015). Analysis of 13 cell types reveals evidence for the expression of numerous novel primate- and tissue-specific microRNAs. Proceedings of the National Academy of Sciences of the United States of America.

[ref-31] Lowery AJ, Miller N, Devaney A, McNeill RE, Davoren PA, Lemetre C, Benes V, Schmidt S, Blake J, Ball G, Kerin MJ (2009). MicroRNA signatures predict oestrogen receptor, progesterone receptor andHER2/neu receptor status in breast cancer. Breast Cancer Research.

[ref-32] Lu Y, Qin B, Hu H, Zhang J, Wang Y, Wang Q, Wang S (2016). Integrative microRNA-gene expression network analysis in genetic hypercalciuric stone-forming rat kidney. PeerJ.

[ref-33] Mattie MD, Benz CC, Bowers J, Sensinger K, Wong L, Scott GK, Fedele V, Ginzinger D, Getts R, Haqq C (2006). Optimized high-throughput microRNA expression profiling provides novel biomarker assessment of clinical prostate and breast cancer biopsies. Molecular Cancer.

[ref-34] McDermott AM, Miller N, Wall D, Martyn LM, Ball G, Sweeney KJ, Kerin MJ (2014). Identification and validation of oncologic miRNA biomarkers for luminal A-like breast cancer. PLOS ONE.

[ref-35] Mortazavi A, Williams BA, McCue K, Schaeffer L, Wold B (2008). Mapping and quantifying mammalian transcriptomes by RNA-Seq. Nature Methods.

[ref-36] Nian W, Ao X, Wu Y, Huang Y, Shao J, Wang Y, Chen Z, Chen F, Wang D (2013). miR-223 functions as a potent tumor suppressor of the Lewis lung carcinoma cell line by targeting insulin-like growth factor-1 receptor and cyclin-dependent kinase 2. Oncology Letters.

[ref-37] Niu Y, Zhou H, Liu Y, Wang Y, Xie J, Feng C, An N (2019). miR-16 regulates proliferation and apoptosis of pituitary adenoma cells by inhibiting HMGA2. Oncology Letters.

[ref-38] Niyazova R, Berillo O, Sh Atambayeva, Pyrkova A, Alybayeva A, Ivashchenko A (2015). miR-1322 binding sites in paralogous and orthologous genes. BioMed Research International.

[ref-39] Patel Y, Shah N, Lee JS, Markoutsa E, Jie C, Liu S, Botbyl R, Reisman D, Xu P, Chen H (2016). A novel double-negative feedback loop between miR-489 and the HER2-SHP2-MAPK signaling axis regulates breast cancer cell proliferation and tumor growth. Oncotarget.

[ref-40] Pearlman A, Rahman M, Upadhyay K, Loke J, Ostrer H (2019). Ectopic Otoconin 90 expression in triple negative breast cancer cell lines is associated with metastasis functions. PLOS ONE.

[ref-41] Persson H, Kvist A, Rego N, Staaf J, Vallon-Christersson J, Luts L, Loman N, Jonsson G, Naya H, Hoglund M, Borg A, Rovira C (2011). Identification of new microRNAs in paired normal and tumor breast tissue suggests a dual role for the ERBB2/Her2 gene. Cancer Research.

[ref-42] Piasecka D, Braun M, Kordek R, Sadej R, Romanska H (2018). MicroRNAs in regulation of triple-negative breast cancer progression. Journal of Cancer Research and Clinical Oncology.

[ref-43] Piscuoglio S, Zlobec I, Pallante P, Sepe R, Esposito F, Zimmermann A, Diamantis I, Terracciano L, Fusco A, Karamitopoulou E (2012). HMGA1 and HMGA2 protein expression correlates with advanced tumour grade and lymph node metastasis in pancreatic adenocarcinoma. Histopathology.

[ref-44] Qian P, Banerjee A, Wu ZS, Zhang X, Wang H, Pandey V, Zhang WJ, Lv XF, Tan S, Lobie PE, Zhu T (2012). Loss of SNAIL regulated miR-128-2 on chromosome 3p22.3 targets multiple stem cell factors to promote transformation of mammary epithelial cells. Cancer Research.

[ref-45] Sun M, Gomes S, Chen P, Frankenberger CA, Sankarasharma D, Chung CH, Chada KK, Rosner MR (2014). RKIP and HMGA2 regulate breast tumor survival and metastasis through lysyl oxidase and syndecan-2. Oncogene.

[ref-46] Sung H, Jeon S, Lee KM, Han S, Song M, Choi JY, Park SK, Yoo KY, Noh DY, Ahn SH, Kang D (2012). Common genetic polymorphisms of microRNA biogenesis pathway genes and breast cancer survival. BMC Cancer.

[ref-47] Tahiri A, Leivonen SK, Lüders T, Steinfeld I, Ragle Aure M, Geisler J, Mäkelä R, Nord S, Riis ML, Yakhini Z, Kleivi Sahlberg K, Børresen-Dale AL, Perälä M, Bukholm IR, Kristensen VN (2014). Deregulation of cancer-related miRNAs is a common event in both benign and malignant human breast tumors. Carcinogenesis.

[ref-48] Takehara M, Nishimura T, Mima S, Hoshino T, Mizushima T (2009). Effect of claudin expression on paracellular permeability, migration and invasion of colonic cancer cells. Biological and Pharmaceutical Bulletin.

[ref-49] Telonis AG, Loher P, Jing Y, Londin E, Rigoutsos I (2015). Beyond the one-locus-one-miRNA paradigm: microRNA isoforms enable deeper insights into breast cancer heterogeneity. Nucleic Acids Research.

[ref-50] Wang S, Huang X, Li Y, Lao H, Zhang Y, Dong H, Xu W, Li JL, Li M (2011a). RN181 suppresses hepatocellular carcinoma growth by inhibition of the ERK/MAPK pathway. Hepatology.

[ref-51] Wang S, Li H, Wang J, Wang D (2013). Expression of microRNA-497 and its prognostic significance in human breast cancer. Diagnostic Pathology.

[ref-52] Wang SE, Lin RJ (2013). MicroRNA and HER2-overexpressing cancer. Microrna.

[ref-53] Wang X, Liu X, Li AY, Chen L, Lai L, Lin HH, Hu S, Yao L, Peng J, Loera S, Xue L, Zhou B, Zhou L, Zheng S, Chu P, Zhang S, Ann DK, Yen Y (2011b). Overexpression of HMGA2 promotes metastasis and impacts survival of colorectal cancers. Clinical Cancer Research.

[ref-54] Wang W, Luo YP (2015). MicroRNAs in breast cancer: oncogene and tumor suppressors with clinical potential. Journal of Zhejiang University Science B.

[ref-55] Wu ZB, Cai L, Lin SJ, Lu JL, Yao Y, Zhou LF (2013). The miR-92b functions as a potential oncogene by targeting on Smad3 in glioblastomas. Brain Research.

[ref-56] Yakovchuk P, Protozanova E, Frank-Kamenetskii MD (2006). Base-stacking and base-pairing contributions into thermal stability of the DNA double helix. Nucleic Acids Research.

[ref-57] Yang S, Sui J, Liang G (2017). Diagnosis value of aberrantly expressed microRNA profiles in lung squamous cell carcinoma: a study based on the Cancer Genome Atlas. PeerJ.

[ref-58] Yang Z, Wu L, Wang A, Tang W, Zhao Y, Zhao H, Teschendorff AE (2017). dbDEMC 2.0: updated database of differentially expressed miRNAs in human cancers. Nucleic Acids Research.

[ref-59] Yao L, Liu Y, Cao Z, Li J, Huang Y, Hu X, Shao Z (2018). MicroRNA-493 is a prognostic factor in triple-negative breast cancer. Cancer Science.

[ref-60] Yu Y, Wu J, Guan L, Qi L, Tang Y, Ma B, Zhan J, Wang Y, Fang W, Zhang H (2013). Kindlin 2 promotes breast cancer invasion via epigenetic silencing of the microRNA200 gene family. International Journal of Cancer.

[ref-61] Yurikova OYu, Aisina DE, Niyazova RE, Atambayeva ShA, Labeit S, Ivashchenko AT (2019). The Interaction of miRNA-5p and miRNA-3p with the mRNAs of Orthologous Genes. Molecular Biology.

[ref-62] Zhang K, Wang YW, Wang YY, Song Y, Zhu J, Si PC, Ma R (2017). Identification of microRNA biomarkers in the blood of breast cancer patients based on microRNA profiling. Gene.

