# Supplementary material for: Prediction of clusters of miRNA binding sites in mRNA candidate genes of breast cancer subtypes
Source: PeerJ. 2019 Nov 13;7:e8049. doi: 10.7717/peerj.8049 (PMC6858813; doi:10.7717/peerj.8049)
Supplement: Figure S1 [file peerj-07-8049-s001.pdf]

<table>
<tr><td>*CBL*; ID01810.3p-miR; 5'UTR; 31; -113; 87; 23
5'-GGC**G**GCGG**C**GG**CG**GC**G**GC**C**GGGA-3'
   ||||||||||||||||||||| |
3'-CCG**U**CGCC**A**CC**AU**CG**U**CG**A**CCAU-5'</td><td>*RAB5A*; ID03229.5p-miR; 5'UTR; 327; -115; 86; 22
5'-G**GC**G**AC**GCCGCCGCCGCC**A**CC**A**-3'
   ||||||||||||||||||||||
3'-C**UA**C**CA**CGGCGGCGGCGG**C**GG**C**-5'</td></tr>
<tr><td>*RUNX1*; ID01321.5p-miR; 5'UTR; 1436; -110; 90; 21
5'-CCCGCCC**CC**CC**C**CA**C**CCCCCG-3'
   |||||||||||| |||||||
3'-GGGCGGG**AA**GG**A**GA**A**GGGGGC-5'</td><td>*CBL*; miR-3198; CDS; 1589; -106; 88; 22
5'-UCUCCAUUC**U**CCAUG**G**C**C**CCAC-3'
   ||||||||||||| ||||||||
3'-AGAGGUAAG**G**GGUCC**U**G**A**GGUG-5'</td></tr>
<tr><td>*ATM*; miR-5095; 3'UTR; 9787; -108; 93; 21
5'-CGC**A**G**C**GG**C**UCACGCCUGUAA-3'
   |||||||||||||||||||||
3'-GCG**C**C**A**CC**A**AGUGCGGACAUU-5'</td><td>*ATM*; miR-619-5p; 3'UTR; 9793; -119; 98; 22
5'-GCCUG**G**CCA**GU**AUGGUGAAAC-3'
   |||||||||||||||||||||
3'-CGGAC**U**GGU**UG**UACCACUUUG-5'</td></tr>
<tr><td>*ATM*; miR-5096; 3'UTR; 9882; -104; 92; 21
5'-GCCUG**G**CCA**GU**AUGGUGAAAC-3'
   |||||||||||||||||||||
3'-CGGAC**U**GGU**UG**UACCACUUUG-5'</td><td>*ATM*; miR-1273a; 3'UTR; 11054; -119; 90; 25
5'-**G**AGACAGAGUCUUGCU**C**UGUC**A**CCC-3'
   |||| ||||||||||||||||||||
3'-**U**UCUUUCUCAGAACGA**A**ACAG**C**GGG-5'</td></tr>
<tr><td>*ATM*; miR-1273g-3p; 3'UTR; 11076; -113; 96; 21
5'-C**C**CAGGCUGGAGUGCAGUGG**C**-3'
   |||||||||||||||||||||
3'-G**A**GUCCGACCUCACGUCACC**A**-5'</td><td>*ATM*; miR-1273e; 3'UTR; 11119; -108; 93; 22
5'-UC**UG**C**C**UCCUGGGUUCAAGCAA-3'
   ||||||||||||||||||||||
3'-AG**GU**G**A**AGGACCCAAGUUCGUU-5'</td></tr>
<tr><td>*CBL*; miR-1273a; 3'UTR; 7727; -117; 89; 25
5'-**G**AGAU**G**GAGUCU**C**GCUGUGUCGCCC-3'
   |||| ||||||||||| ||||||||
3'-**U**UCUU**U**CUCAGA**A**CGAAACAGCGGG-5'</td><td>*CBL*; miR-566; 3'UTR; 7838; -98; 90; 19
5'-G**C**UGGGAU**U**ACAGGCGCC**U**-3'
   |||||||||||||||||||
3'-C**A**ACCCUA**G**UGUCCGCGG**G**-5'</td></tr>
<tr><td>*CBL*; miR-3155a; 3'UTR; 10588; -106; 91; 21
5'-AGUGCCCUCUGCAG**G**GCCUGG-3'
   ||| ||| |||||||||||||
3'-UCAAGGGUGACGUC**U**CGGACC-5'</td><td>*IL11*; miR-1273f; 3'UTR; 1466; -102; 98; 19
5'-CACUGCAACCUCCA**C**CUCC-3'
   |||||||||||||||||||
3'-GUGACGUUGGAGGU**A**GAGG-5'</td></tr>
<tr><td>*IL11*; miR-1273d; 3'UTR; 1467; -121; 89; 25
5'-ACUGCA**A**CCUCCACCUCC**C**GGGUUC-3'
   ||||||||||| ||||| |||||||
3'-UGACGU**C**GGAGUUGGAGU**A**CCCAAG-5'</td><td>*IL11*; miR-1273e; 3'UTR; 1476; -113; 96; 22
5'-UCCAC**C**UCC**C**GGGUUCAAGCAA-3'
   ||||||||||||||||||||||
3'-AGGUG**A**AGG**A**CCCAAGUUCGUU-5'</td></tr>
<tr><td>*IL11*; ID01404.5p-miR; 3'UTR; 1476; -113; 91; 23
5'-GCAA**C**CUC**C**ACCUC**C**CGGGUUCA-3'
   ||||||||| |||||||||||||
3'-CGUU**A**GAG**A**AGGAG**A**GCCCAAGU-5'</td><td>*IL11*; miR-5095; 3'UTR; 1982; -106; 91; 21
5'-C**AU**GGUGG**C**UCACGCCUGUAA-3'
   |||||||||||||||||||||
3'-G**CG**CCACC**A**AGUGCGGACAUU-5'</td></tr>
<tr><td>*RUNX1*; ID00436.3p-miR; 3'UTR; 5464; -108; 93; 23
5'-GUGUGUGCGU**G**U**G**U**G**UGUGUGU**G**-3'
   |||||||||||||||||||||||
3'-CACACACGCA**U**A**U**A**U**ACACACA**U**-5'</td><td>*STMN1*; miR-1273a; 1729; 3'UTR; -115; 87; 25
5'-**G**AG**G**CAGAGUCU**CA**CUCUGUCGCCC-3'
   |||| ||||||||||||||||||||
3'-**U**UC**U**UUCUCAGA**AC**GAAACAGCGGG-5'</td></tr>
<tr><td>*STMN1*; miR-1273c; 1731; 3'UTR; -106; 88; 22
5'-G**G**CAG**A**GUCUC**AC**U**C**UGUCGCC-3'
   ||||||||||||||||||||||
3'-C**U**GUC**C**CAGAG**CA**A**A**ACAGCGG-5'</td><td>*STMN1*; miR-1273-3p; 1751; 3'UTR; -103; 93; 21
5'-C**C**CAGGCUGGAGGGCAGUGG**C**-3'
   |||||||||||| ||||||||
3'-G**A**GUCCGACCUCACGUCACC**A**-5'</td></tr>
<tr><td>*STMN1*; miR-1285-3p; 1735; 3'UTR; -104; 89; 22
5'-**GA**GUCUCACU**C**UGU**C**GCCCAG**G**-3'
   ||||||||||||||||||||||
3'-**UC**CAGAGUGA**A**ACA**A**CGGGUC**U**-5'</td><td>*STMN1*; miR-5585-3p; 1831; 3'UTR; -106; 91; 22
5'-CUC**C**CGAGUAGCUGGGACUACA-3'
   ||||||||||||||| ||| ||
3'-GAG**A**GCUCAUCGACCAUGAAGU-5'</td></tr>
<tr><td>*SFN*; ID00436.3p-miR; 3'UTR; 1190; -104; 89; 23
5'-GUGUGUG**U**GU**G**U**G**U**G**UGUGUGU**G**-3'
   |||||||||||||||||||||||
3'-CACACAC**G**CA**U**A**U**A**U**ACACACA**U**-5'</td><td>*SFN*; ID00436.3p-miR; 3'UTR; 1202; -104; 89; 23
5'-GUGUGUG**U**GU**G**U**G**U**G**UGUGUGU**G**-3'
   |||||||||||||||||||||||
3'-CACACAC**G**CA**U**A**U**A**U**ACACACA**U**-5'</td></tr>
<tr><td>*SFN*; ID01727.5p-miR; 3'UTR; 1203; -106; 91; 23
5'-UGUGUGUGUGUGUGU**G**UGUGUGC-3'
   ||||||| ||| |||||||||||
3'-ACACACAAACAAACA**U**ACACACG-5'</td><td>*SFN*; ID02868.3p-miR; 3'UTR; 1188; -113; 90; 23
5'-**G**GGUGUGUGUGUGUGUGUGUG**U**G-3'
   |||||| ||||||||||||||||
3'-**U**CCACAGACACACACACACAC**G**C-5'</td></tr>
<tr><td colspan="2">Note: Gene; miRNA; the miRNA region; start of binding site (nt); the free energy, ΔG (kJ/mole); the ΔG/ΔGm (%); length of miRNA (nt). The upper and lower nucleotide sequences of mRNA and miRNA, respectively. The nucleotides of non-canonical pairs G-U and A-C highlighted in bold type.</td></tr>
</table>

Supplemental Figure S1 Schemes of miRNA interaction with mRNA of candidate genes of breast cancer triple-negative subtype.
