# Supplementary material for: Prediction of clusters of miRNA binding sites in mRNA candidate genes of breast cancer subtypes
Source: PeerJ. 2019 Nov 13;7:e8049. doi: 10.7717/peerj.8049 (PMC6858813; doi:10.7717/peerj.8049)
Supplement: Figure S2 [file peerj-07-8049-s002.pdf]

<table>
<tr><td>FOXA1; ID03367.5p-miR; 5'UTR; 122; -117; 93; 20
5'-CCGCGCCGCCGCCGCC**G**CCG-3'
|||||||||||||||||||
3'-CGCGCGGCGGCGGCGG**U**GGC-5'</td><td>HMGA2; ID01403.5p-miR; 5UTR; 547; -119; 87; 23
5'-C**A**C**C**CACCGC**C**GC**C**GCCGCC**A**C**C**-3'
|||||||||||||||||||||||
3'-G**C**G**A**GUGGCG**A**CG**A**CGGCGG**C**G**A**-5'</td></tr>
<tr><td>HMGA2; ID01641.3p-miR; 5UTR; 545; -132; 89; 24
5'-CCCACCCACCGCCGCCGCCGCCAC-3'
||| || ||||||||||||||||
3'-GGGGUGGGGGCGGCGGCGGCGGUG-5'</td><td>HMGA2; ID02428.3p-miR; 5UTR; 315; -108; 88; 22
5'-GGUGGGGGG**A**A**GAG**GA**G**GAGGA-3'
||||||||||||||||||||||
3'-CCACCCCCC**C**U**UCU**CU**U**CUCCU-5'</td></tr>
<tr><td>HMGA2; ID03324.3p-miR; 5UTR; 312; -110; 87; 22
5'-CAGGGUGGG**G**GGA**A**GAGGAGG**A**-3'
|||||||||||||||||||||
3'-CUCCCACCC**U**CCU**C**CUCCUCC**C**-5'</td><td>SMAD3; miR-4508; 5'UTR; 111; -100; 94; 17
5'-CGCGCGCCGAGCCCCGC-3'
|||||||| ||||||||
3'-GCGCGCGGGUCGGGGCG-5'</td></tr>
<tr><td>FOXA1; ID02542.5p-miR; CDS; 1129; -125; 87; 24
5'-GG**G**GCCGGCGGCGG**G**GGCG**G**G**A**G**C**-3'
|||||||||||||||| |||||||
3'-CC**U**CGGCCGCCGCC**U**CGGC**U**C**C**C**A**-5'</td><td>FOXA1; ID03332.3p-miR; CDS; 1150; -134; 90; 24
5'-**A**GCGGA**A**GCGG**G**GGC**A**GCGGCGCC-3'
||||||||||||||||||||||||
3'-**C**CGCCU**C**CGCC**U**CCG**C**CGCCGCGG-5'</td></tr>
<tr><td>HMGA2; ID00101.3p-miR; 3'UTR; 1263; -110; 88; 22
5'-GGUGGGGUGGGGGAG**GG**G**GG**GG-3'
| ||||||||||||||||||||
3'-CGACCCCACCCCCUC**UU**C**UU**CC-5'</td><td>HMGA2; ID00849.3p-miR; 3'UTR; 1268; -117; 90; 22
5'-GGUGGG**G**GAGGGGG**G**G**G**UGGGG-3'
||||||||||||||||| ||||
3'-CCACCC**U**CUCCCCC**U**C**U**CCCCC-5'</td></tr>
<tr><td>SMAD3; miR-4507; 3'UTR; 2066; -106; 91; 20
5'-CCCAGCCCAGCCCC**G**CCCCG-3'
||||||||||||| |||| |
3'-GGGUCGGGUCGGGU**U**GGGUC-5'</td><td>SMAD3; miR-937-5p; 3'UTR; 2072; -102; 89; 20
5'-CCAGCCCC**G**CCC**C**GCC**C**C**G**C-3'
|||||||||||||| |||||
3'-GGUCGGGG**U**GGG**A**CUG**A**G**U**G-5</td></tr>
<tr><td>TGFB1; miR-877-3p; 233; 5'UTR; -108; 93; 21
5'-CGGGGAGGAGGG**G**GA**G**GAGGA-3'
| |||||||||||||||||||
3'-GACCCUCCUCCC**U**CU**U**CUCCU-5'</td><td>TGFB1; miR-937-5p; 2089; 3'UTR; -102; 89; 20
5'-CC**G**GCCCCACCC**C**GCCCC**G**C-3'
|||||||||||||| |||||
3'-GG**U**CGGGGUGGG**A**CUGAG**U**G-5'</td></tr>
<tr><td>HMGA2; ID01403.5p-miR; 5'UTR; 547; -119; 88; 23
5'-C**A**C**C**CACCGC**C**GC**C**GCCGCC**A**C**C**-3'
|||||||||||||||||||||||
3'-G**C**G**A**GUGGCG**A**CG**A**CGGCGG**C**G**A**-5'</td><td>SMAD3; ID01020.5p-miR; 5'UTR; 194; -117; 100; 19
5'-GCGACCGCGGCAGGCCCCG-3'
|||||||||||||||||||
3'-CGCUGGCGCCGUCCGGGGC-5'</td></tr>
<tr><td>ANGPTL4; ID01593.5p-miR; CDS; 259; -134; 100; 23
5'-AGCGCUCAGGGCGGACCCGUGCA-3
|||||||||||||||||||||||
3'-UCGCGAGUCCCGCCUGGGCACGU-5'</td><td>FOXA1; ID03332.3p-miR; CDS; 150; -134; 90; 24
5'-**A**GCGGA**A**GCGG**G**GGC**A**GCGGCGCC-3'
||||||||||||||||||||||||
3'-**C**CGCCU**C**CGCC**U**CCG**C**CGCCGCGG-5'</td></tr>
<tr><td colspan="2">Note: Gene; miRNA; the miRNA region; start of binding site (nt); the free energy, ΔG (kJ/mole); the ΔG/ΔGm (%); length of miRNA (nt). The upper and lower nucleotide sequences of mRNA and miRNA, respectively. The nucleotides of non-canonical pairs G-U and A-C highlighted in bold type.</td></tr>
</table>

Supplemental Figure S2 Schemes of miRNA interaction with mRNA of candidate genes of breast cancer luminal A and B subtypes.
