# Supplementary material for: Prediction of clusters of miRNA binding sites in mRNA candidate genes of breast cancer subtypes
Source: PeerJ. 2019 Nov 13;7:e8049. doi: 10.7717/peerj.8049 (PMC6858813; doi:10.7717/peerj.8049)
Supplement: Figure S3 [file peerj-07-8049-s003.pdf]

<table>
<tr><td>*MAZ*; ID02998.3p-miR; 5'UTR; 22; -113; 90; 21
5'-**G**GUGCGC**G**GGCGGC**G**GGGCGG-3'
||||||||||||| |||||||
3'-**U**CACGCG**U**CCGCCC**U**CCCGCC-5'</td><td>*MAZ*; ID02979.5p-miR; 5'UTR; 114; -121; 92; 22
5'-CG**G**CC**C**GCGCCCCC**G**GCCCCCG-3'
||||||||||||||||||||||
3'-GC**U**GG**A**CGCGGGGG**U**CGGGGGA-5'</td></tr>
<tr><td>*MAZ*; miR-5008-5p; 5'UTR; 133; -110; 88; 22
5'-CC**G**CUGAGCCCC**GG**GGGCC**C**C**G**-3'
|||||| |||||||||||||||
3'-GG**U**GACACGGGG**UU**CCCGG**A**G**U**-5'</td><td>*NISCH;* ID01996.3p-miR; 5'UTR; 34; -110; 87; 21
5'-GGGG**G**CG**A**CG**G**GCGGCGGGG**G**-3'
|||||||| ||||||||||||
3'-CCCC**U**GC**C**CC**U**CGCCGCCCC**U**-5'</td></tr>
<tr><td>*MAZ*; ID02499.3p-miR; CDS; 488; -115; 89; 21
5'-CCGCCG**U**CGC**U**GCC**G**CGCCCC-3'
||||||||||||||||| |||
3'-GGCGGC**G**GCG**G**CGG**U**GCUGGG-5'</td><td>*MAPK3*; miR-6805-3p; CDS; 1145; -117; 87; 23
5'-CUGGGGGC**A**GGGGAGCAG**G**GG**GG**-3'
||||||||||||||||||||| ||
3'-GACCCCCG**C**CCCCUCGUC**U**CG**UU**-5'</td></tr>
<tr><td>*MAZ*; miR-3960; CDS; 506; -113; 90; 20
5'-CCCCGGCC**C**C**U**GCCGCCGCC-3'
|||| |||||||||||||||
3'-GGGGGCGG**A**G**G**CGGCGGCGG-5'</td><td>*CDK6*; ID00436.3p-miR; 3'UTR; 1896; -104; 89; 23
5'-GUGUGUG**U**GU**GC**AU**G**UGUGUGU**G**-3'
|||||||||||||||||||||||
3'-CACACAC**G**CA**UA**UA**U**ACACACA**U**-5'</td></tr>
<tr><td>*MAZ*; miR-3960; CDS; 614; -117; 93; 20
5'-CCCCCGCCUCCGCCGCC**A**C**U**-3'
||||||||||||||||||||
3'-GGGGGCGGAGGCGGCGG**C**G**G**-5'</td><td>*MAZ*; miR-6729-5p; CDS; 361; -115; 87; 22
5'-GCCGCGCCGGCGCCC**C**CGCCCA-3'
||||| | | ||||||||||||
3'-CGGCGAGUCGGCGGG**A**GCGGGU-5'</td></tr>
<tr><td>*NISCH*; miR-877-3p; CDS; 2141; -102; 87; 21
5'-C**CA**GG**G**GGAGG**A**AGA**G**GAGGA-3'
|||||||||||||||||||||
3'-G**AC**CC**U**CCUCC**C**UCU**U**CUCCU-5'</td><td>*MAZ*; miR-877-3p; 3'UTR; 2273; -106; 91; 21
5'-C**CA**GG**G**GGAGGGAG**G**AGAGGA-3'
|||||||||||||||||||||
3'-G**AC**CC**U**CCUCCCUC**U**UCUCCU-5'</td></tr>
<tr><td>*MAZ*; miR-7111-3p; 3'UTR; 2273; -106; 88; 22
5'-C**CA**GG**G**GGAGGGA**G**GAGAGGAA-3'
||||||||||||||||||||||
3'-G**AC**CC**U**CCUCCCU**U**CUCUCCUA-5'</td><td>*MAZ*; ID01352.3p-miR; 3'UTR; 2274; -110; 87; 23
5'-**C**AGGG**G**G**A**G**G**GAGGAG**AG**GAAGG-3'
|||||||||||||||||||||||
3'-**A**UCCC**U**C**C**C**U**CUCCUC**CU**CUUCC-5'</td></tr>
<tr><td colspan="2">Note: Gene; miRNA; the miRNA region; start of binding site (nt); the free energy, ΔG (kJ/mole); the ΔG/ΔGm (%); length of miRNA (nt). The upper and lower nucleotide sequences of mRNA and miRNA, respectively. The nucleotides of non-canonical pairs G-U and A-C highlighted in bold type.</td></tr>
</table>

Supplemental Figure S3 Schemes of miRNA interaction with mRNA of candidate genes of breast cancer HER2 subtype.
