# Supplementary material for: Prediction of clusters of miRNA binding sites in mRNA candidate genes of breast cancer subtypes
Source: PeerJ. 2019 Nov 13;7:e8049. doi: 10.7717/peerj.8049 (PMC6858813; doi:10.7717/peerj.8049)
Supplement: Table S1 [file peerj-07-8049-s004.pdf]

**Supplemental Table S1 Candidate genes of subtypes of breast cancer, indicating sources of information on their participation in oncogenesis of breast cancer.**

| Gene (ID) | A source of information | Gene (ID) | A source of information |
|---|---|---|---|
| Subtype triple-negative (basal-like) | | Subtype HER2 | |
| *ATM* (ID: 472) | PMID: 29063517 | *ADAM17* (ID: 6868) | PMID: 21501859 |
| *AXL* (ID: 558) | PMID: 29097911 | *AURKA* (ID: 6790) | PMID: 29214214 |
| *BIRC5* (ID: 332) | PMID: 22713668 | *BRCA2* (ID: 675) | PMID: 27195161 |
| *CBL* (ID: 867) | PMID: 23784775 | *BRIP1* (ID: 83990) | PMID: 26824983 |
| *CD44* (ID: 960) | PMID: 29301020 | *CDK6* (ID: 1021) | PMID: 29263697 |
| *CEACAM5* (ID: 1048) | PMID: 28457854 | *EPOR* (ID: 2057) | PMID: 23117856 |
| *ERBB3* (ID: 2065) | PMID: 27845906 | *ERBB3* (ID: 2065) | PMID: 29262628 |
| *F2RL1* (ID: 2150) | PMID: 29195005 | *H2AFX* (ID: 3014) | PMID: 25252808 |
| *FGFR2* (ID: 2263) | PMID: 27220763 | *MAPK3* (ID: 5595) | PMID: 28579432 |
| *FIS1* (ID: 51024) | PMID: 24355041 | *MAZ* (ID: 4150) | PMID: 22102859 |
| *IAPP* (ID: 3375) | PMID: 29108265 | *NISCH* (ID: 11188) | PMID: 28363452 |
| *IL11* (ID: 3589) | PMID: 22629385 | *TIMP3* (ID: 7078) | PMID: 22520950 |
| *JHDM1D* (ID: 80853) | PMID: 22573479 | Subtypes luminal A and B | |
| *LAMC1* (ID: 3915) | PMID: 22578566 | *ANGPTL4* (ID: 51129) | PMID: 23354167 |
| *LASP1* (ID: 3927) | PMID: 22713668 | *EZH1* (ID: 2145) | PMID: 22685420 |
| *MAGEA10* (ID: 4109) | PMID: 24560456 | *FOXA1* (ID: 3169) | PMID: 28884749 |
| *MID1* (ID: 4281) | PMID: 23623609 | *GTF2IRD1* (ID: 9569) | PMID: 20348243 |
| *MMP2* (ID: 4313) | PMID: 27340107 | *HMGA2* (ID: 8091) | PMID: 22685420 |
| *PFN1* (ID: 5216) | PMID: 28699810 | *ITGB1* (ID: 3688) | PMID: 27624535 |
| *PRKCE* (ID: 5581) | PMID: 23542175 | *MAPT* (ID: 4137) | PMID: 23262785 |
| *PRRT2* (ID: 112476) | PMID: 29195005 | *MCM7* (ID: 4176) | PMID: 22685420 |
| *RUNX1* (ID: 861) | PMID: 27428424 | *SMAD3* (ID: 4088) | PMID: 24235142 |
| *SERPINE1* (ID: 5054) | PMID: 23721519 | *SOX4* (ID: 6659) | PMID: 22685420 |
| *SFN* (ID: 2810) | PMID: 24067649 | *TGFB1* (ID: 7040) | PMID: 23354167 |
| *STMN1* (ID: 3925) | PMID: 28766688 | | |
