# Supplementary material for: Prediction of clusters of miRNA binding sites in mRNA candidate genes of breast cancer subtypes
Source: PeerJ. 2019 Nov 13;7:e8049. doi: 10.7717/peerj.8049 (PMC6858813; doi:10.7717/peerj.8049)
Supplement: Table S2 [file peerj-07-8049-s005.pdf]

**Supplemental Table S2 Information on miRNAs interacted with genes of breast cancer and other cancer.**

| miRNA | Information about miRNAs | A source of information |
|---|---|---|
| TJU_CMC_MD2.ID03306.3p-miR | MDA-MB-231 cells (BC) | PMID: 26400174 |
| miR-127-3p | Exosomes (BC) | PMID: 30309355 |
| miR-466 | Prostate cancer cells | PMID: 28125091 |
| miR-548aq-3p | Intrahepatic cholangiocarcinoma cells | PMID: 27619971 |
| miR-548z | Hepatoblastoma cells | PMID: 29404451 |
| miR-566 | Glioma cell lines, melanoma cells | PMID: 24650032, PMID: 30323971 |
| miR-574-3p | Melanoma cells, serum (Pancreatic cancer) | PMID: 20302635, PMID: 25664025 |
| miR-574-5p | BC cells | PMID: 28789418 |
| miR-619-5p | Colorectal carcinoma cells | PMID: 28101234 |
| miR-762 | Serum (Pancreatic cancer) | PMID: 25664025 |
| miR-877-3p | Bladder cancer cells | PMID: 27429046 |
| miR-937-5p | BC cells | PMID: 26378051 |
| miR-1273c | BC cells | PMID: 25471792 |
| miR-1273d | Glioma cells | PMID: 26892862 |
| miR-1273e | Urinary exosomes | PMID: 29479137 |
| miR-1273f | Plasma (hepatocellular carcinoma) | PMID: 28657540 |
| miR-1273-3p | Colon cancer cells | PMID: 30186855 |
| miR-1273g-3p | Serum (ovarian cancer) | PMID: 30264202 |
| miR-1285-3p | Plasma (hepatocellular carcinoma) | PMID: 25230788 |
| miR-1470 | Serum (esophageal squamous cell carcinoma) | PMID: 29344220 |
| miR-1908-3p | Mutant UQCRB-expressing cells (cancer) | PMID: 30120311 |
| miR-2861 | Cervical cancer tissues | PMID: 27364926, PMID: 26656154 |
| miR-3155a | Prostate, bladder cancer cells | PMID: 29291244 |
| miR-3198 | Stem cell, serum (pancreatic cancer) | PMID: 30389909, PMID: 25664025 |
| miR-3620-5p | Glioma-associated human mesenchymal cells | PMID: 28855213 |
| miR-3960 | BC cells | PMID: 28886127 |
| miR-4466 | BC cells | PMID: 28789418 |
| miR-4507-3p | BC cells | PMID: 26397135 |
| miR-4508 | BC cells | PMID: 27019627 |
| miR-4690-5p | Exosomes (hyperhomocysteinemia) | PMID: 29560091 |
| miR-4706 | Melanoma cells | PMID: 26778792 |
| miR-4787-5p | Pancreatic cancer cells | PMID: 27624777 |
| miR-5008-5p | Gastric cancer | PMID: 30373600 |
| miR-5095 | Cholangiocarcinoma cells | PMID: 29620172 |
| miR-5096 | Glioma cells | PMID: 25978028 |
| miR-5585-3p | BC cells | PMID: 28789418 |

| | | |
|---|---|---|
| miR-6089 | Colorectal cancer cells | PMID: 27780077 |
| miR-6729-5p | Exosomes (aging) | PMID: 26370963 |
| miR-6756-5p | BC cells | PMID: 29607263 |
| miR-6805-3p | Oral mucosal melanoma cells | PMID: 30323971 |
| miR-6805-5p | Oral mucosal melanoma cells | PMID: 30323971 |
| miR-6846-5p | Oral mucosal melanoma cells | PMID: 30323971 |
| miR-6850-5p | Excretory system cancer cells | PMID: 27019673 |
| miR-7111-3p | Exosomes (aging) | PMID: 26370963 |
