# Supplementary material for: Prediction of clusters of miRNA binding sites in mRNA candidate genes of breast cancer subtypes
Source: PeerJ. 2019 Nov 13;7:e8049. doi: 10.7717/peerj.8049 (PMC6858813; doi:10.7717/peerj.8049)
Supplement: Table S3 [file peerj-07-8049-s006.pdf]

Supplemental Table S3 The nucleotide sequence of clusters of miRNA binding sites in orthologous candidate genes of triple-negative subtype.

Gene	Nucleotide sequences of cluster	Species
CBL	ggcggcggcggcggcggcggcggcggcggccgggag	Hsa
	gcccggauagccggcggcggcggcggcggccgggag	Ptr
	gcccggauagccggcggcggcggcggcggccgggag	Ggo
	gcccggauagccggcggcggcggcggccgggaggag	Pan
*CBL***	gagauggagucucgcugugucgcccaggcuggag	Hsa
	gagacggagucucgcugugucgcccaggcuggag	Ggo
	gagacagagucucgcugugucgcccaggcuggag	Ptr
	gagacagagucucgcugugucgcccaggcuggag	Ppa
	gagaaggagucuugcugugucaccuaggcuggag	Pan
MMP2	gcggcggcggcggcggcggcgggggcuggggcgcgggg	Hsa
	gcggcggcggcggcggcggcgggggcuggggagcgggg	Pab
	gcggcggcggcggcggcggcgggggcuggggcgcggag	Ggo
	gcggcggcggcggcggcggcgggggcuggggcgcgggc	Ptr
	gcggcggcggcggcggcggcagcgggggcgcgggggcc	Mml
	gcggcggcggcggcggcggcggcggcggcgggggcgcg	Pan
RAB5A	ggcggcggaggaggagaaaggaaagaggaa	Hsa
	ggcggcggaggaggagaaaggaaagaggaa	Ptr
	ugcggcggaggaggggaaaggaaagaggaa	Mml
	ggcggcggaggaggagaaaggaaagaggaa	Pab
	ucuggaggaggaggagaaaggaaggcggaa	Mmu
RAB5A	gccaccaccaccgccauagauacacuc	Hsa
	gccaccaccaccgccauagauacacuc	Ptr
	gccaccaccaccgccauagauacacuc	Pab
	gccaccaccaccgccauagacacacac	Mml
*ATM***	cgggcugggcgcagcggcucacgccuguaaucccagc	Hsa
	ugggcugggcgcagcagcucacgccuguaaucccagc	Pab
	cgggcugggcgcagcggcucacgccuguaaucccagc	Ptr
	cgggcuggauacagcagcucacgccuguaaucccggc	Pan
	cgggcugggcgcagcggcucacgccuguaaucccagc	Ppa
*ATM***	uaaugagacagagucuugcucugucacccaggcuggagugcaguggc	Hsa
	uaaugagacagagucuugcucugucacccaggcuggagugcaguggc	Ptr
	aguagagacagaguuucaccguguuagccaggauggucucgaucgcu	Ppa
	aguagagacagaguuucaccguguuagccaggauggucucgaucgcu	Pan
	aguagagacugaguuucaccguguuagccaggauggucuccaucgcc	Pab
*IL11***	cacugcaaccuccaccucccggguucaagcaa	Hsa
	cacugcaaccuccaccucccggguucaagcaa	Ptr
	cacugcaaccuccaccucccggguucaagcaa	Ppa
*RUNX1***	guaugugugugugugcgugugugugugugug	Hsa
	gugugugugugugugcgugugugugugugugug	Ptr
	gugugugugugugugugugugugugugauaugugugug	Mml
	uuuugugugugugugugugugugugugugugagag	Pan
*SFN***	ccgccaccgccccgcccugccccuuccaguccccc	Ptr
	ccgccaccgccccgcccugcccccuccaguccccc	Hsa
	ccgccaccgccccgcccugcccccuccaguccccc	Ggo
	ccgccaccgccccgcccugcccccuccaguccccc	Ppa
*SFN***	gcuggagaugggugugugugugugugugugugugugugugugugugcgcgcg	Hsa
	gcuggagaugggugugugugugugugugugugugugugugugugugugacag	Ptr
	gcuggagaugggugugugugugugugugugugugagugugugcgcgcgcgcg	Ppa
*STMN1***	gaggcagagucucacucugucgcccaggcuggagggcagugg	Hsa
	gaggcagagucucacucugucgcccaggcuggagggcagugg	Ppa
Note. In Tables S3, S4 and S5 shown: Genes without * - miRNA binding sites are in the 5'UTR, genes with * - miRNA binding sites are in the CDS, ** - miRNA binding sites are in the 3'UTR.		
