# Supplementary material for: Prediction of clusters of miRNA binding sites in mRNA candidate genes of breast cancer subtypes
Source: PeerJ. 2019 Nov 13;7:e8049. doi: 10.7717/peerj.8049 (PMC6858813; doi:10.7717/peerj.8049)
Supplement: Table S4 [file peerj-07-8049-s007.pdf]

Supplemental Table S4 The nucleotide sequence of clusters of miRNA binding sites in orthologous candidate genes of luminal A and B subtypes.

Gene	Nucleotide sequence of cluster	Species
FOXA1	cccccggcgcugcccccgcccgccgcgccgccgccgccgccgcgcacgccgc	*Hsa*
	cccccggcgcugcccccucccgccgcgccgcgccgccgccgccgcgcacgcc	*Mml*
	uccccggcgcugcccccucccgccgcgccgcgccgccgccgccgcgcacgcc	*Mmu*
HMGA2	cccaccgccgccgccgccaccggcagcgccuccuccucuccuccuccuccucccccucu	*Hsa*
	cccaccgccgccgccgccaccggcagcgccuccuccucuccuccuccuccucccccucu	*Ptr*
	gccgccgccgccgccgccaccggcagcgccuccuccucuccuccuccuccucccccucu	*Pan*
	gccgccgccgccgccgccaccggcagcgccuccucccccuccuccuccuccucccccucu	*Pab*
	gccgccgccgccgcagccaccggcagcgccuccuccuccucuccuccuccuccuccccu	*Mml*
*HMGA2***	ugggguggggugggguggggagggg-gggggugggguggggagaa	*Hsa*
	ugggguggggugggguggggagggg-gggggugggguggggagaa	*Ptr*
	ugggguggggugggggcgggggagggugggggugggguggggagaa	*Mml*
	ugggguggg-ugggguguggggagggugggggugggguggggagaa	*Pab*
*ITGB1**	ggaggagccgccgccacccgccgcgcccga	*Hsa*
	ggaggagccgccgccacccgccgcgcccga	*Mml*
	ggaggagccgccgccacccgccgcgcccga	*Ptr*
	ggaggagccgccgccacccgccgcgcccga	*Pan*
*SMAD3***	cccagcccagccccgccccgccccgcccc	*Hsa*
	cccagcccggcccagccccgccccgcccc	*Ptr*
	cccagcccggcccagccccgccccgcccc	*Ppa*
	cccagcccagccccagcacuccagcagacc	*Pan*
	cccagcccagccccaccacuccagcagacc	*Pab*
	cccagccccgccccaccacuccagcagacc	*Ggo*
*SOX4***	agcgggagcuaggggcgggggcggaggag	*Hsa*
	agcgggagcuaggggcgggggcggagaag	*Pab*
	agcgggagcuaggggcgggggcggagaag	*Ptr*
	agcgggagcuaggggcgggggcggagaag	*Pan*
*TGFB1***	ccgccccgccccgccccgccccggcaggcccggccccaccccgccccgcccccgcugc	*Hsa*
	ccgccccgccccgccccaccccggcaggcccggccccgccccgccccgcccccgcugc	*Pan*
	ccgccccgccccgccccgccccggcaggcccggccccaccccgcccccgcugccuugc	*Ptr*
	ccgccccgccccgccccgccccggcaggcccggccccaccccgccccgcccugccccc	*Pab*
	ccgccccgccccgccccu-cccggcaggcccggccccgcccccgccccgcccgcagc	*Mmu*
