# Supplementary material for: Prediction of clusters of miRNA binding sites in mRNA candidate genes of breast cancer subtypes
Source: PeerJ. 2019 Nov 13;7:e8049. doi: 10.7717/peerj.8049 (PMC6858813; doi:10.7717/peerj.8049)
Supplement: Table S5 [file peerj-07-8049-s008.pdf]

Supplemental Table S5 **The nucleotide sequence of clusters of miRNA binding sites in orthologous candidate genes of HER2 subtype.**

Gene	Nucleotide sequence of cluster	Species
EPOR	gccgggggcgggggacggaggggcag<br>gccaggggcgggggacggaggggcag<br>gccaggggcgggggacagaggggcag<br>gccaggggcgggggacggaggggcag<br>gccaggggcgggggacagaggggcag<br>gccaggggcgggggacggaggggcag<br>gccaggggcgggggacggaggggcag	*Hsa*<br>*Ptr*<br>*Mml*<br>*Ggo*<br>*Pan*<br>*Ppa*<br>*Pab*
MAZ	ggccggggugcgcgggcggcggggcggcccgcgg<br>ggccggggugcgcgggcggcggggcggcccgcgg<br>ggccggggugcgcgggcggcggggcggcccgcgg	*Hsa*<br>*Ptr*<br>*Pab*
MAZ	cgccccgagcccgggccccgcgcggcccgcgcccccggcccccg<br>cgccccgagcccgggccccgcgcggccagcgcccccggcccccg<br>cgccccgagcccgggccccgcgcggcccgcgcccccggcccccg	*Hsa*<br>*Ptr*<br>*Pab*
*MAZ**	gccgcgccggcgcccccgcccacgccccaggcc<br>gccgcgccggcgcccccgcccacgccccaggcc<br>gccgcgccagcgcccccgcccacgccccaggcc	*Hsa*<br>*Ptr*<br>*Pab*
*MAZ**	gcggcugcugcggccgcugccgccgcugcugccgccgucgcugccgcgcccccggccccugccgccgccucua<br>gcggcugcugcggccgcugccgccgcugcugccgccgucgcugccgcgcccccggccccugcugccgccucca<br>gcggcugcugcggccgcugccgccgcugcugccgccgucgcugccgcgcccccggccccugccgccgccucca	*Hsa*<br>*Ptr*<br>*Pab*
*MAZ**	aggccgcgcccccccgccuccgccgcca<br>aggccgcgcccccccgccuccgccgcca<br>aggccgcgcccccccgccuccgccgcca	*Hsa*<br>*Ptr*<br>*Pab*
*MAZ**	gcggagccggcgggggagggggagaggcgg<br>gcggagccggcgggggagggggagaggcgg<br>gcggagccggcgggggagggggagaggcgg	*Hsa*<br>*Ptr*<br>*Pab*
NISCH	ggcgggggcgacgggcggcgggggccgggggggcg<br>ggcgggggcgucgggcggcgggggcugggggggcg<br>ggcgggggcgaggagcggcgggggcugcggggcg<br>ggcgggggcguggggcggcgggggcugggaggcg<br>ggcgggggcguggggcggcgggggcugggggggcg<br>ggcgggggcgucgggcggcgggggcuggggggcgg	*Hsa*<br>*Ptr*<br>*Pab*<br>*Ggo*<br>*Pan*<br>*Ppa*
*MAPK3**	ccaauaaacggaucacaguggagg<br>ccaauaaacggaucacaguggagg<br>ccaauaaacggaucacaguggagg<br>ccaauaaacggaucacaguggagg	*Hsa*<br>*Ptr*<br>*Ggo*<br>*Pab*
*BRCA2***	aaaacaucuuuggcugagcucgguggcucaugccuguaaucccaac<br>aaaacaucuuuggcugagcucgguggcucaugccuguaaucccaac<br>aaaacaucuuuggcugagcucgguggcucaugccuguaaucccaac<br>aaaacaucuuuggcugagcucaguggcucaugccuguaaucccaac	*Hsa*<br>*Ppa*<br>*Ggo*<br>*Pab*
*CDK6***	ugcaagagugauugcagcuuuauguucauuugu<br>ugcaagagugauugcagcuuuauguucauuugu<br>ugcaagagugauugcagcuuuauguucauuugu<br>ugcaagagugauugcagcuuuauguucauuugu	*Hsa*<br>*Ptr*<br>*Pab*<br>*Ppa*
*CDK6***	ugugugugugcaugugugugugugugugugugugugugugugguaugugag<br>ugugugcgugugugugugugugugugugugugugugugugugguaugugag	*Hsa*<br>*Ptr*
